# The oral bacteriomes of patients with allergic rhinitis and asthma differ from that of healthy controls

**DOI:** 10.3389/fmicb.2023.1197135

**Published:** 2023-06-07

**Authors:** Marcos Pérez-Losada, Eduardo Castro-Nallar, José Laerte Boechat, Luís Delgado, Tiago Azenha Rama, Valentín Berrios-Farías, Manuela Oliveira

**Affiliations:** ^1^Department of Biostatistics and Bioinformatics, Computational Biology Institute, Milken Institute School of Public Health, The George Washington University, Washington, DC, United States; ^2^CIBIO-InBIO, Centro de Investigação em Biodiversidade e Recursos Genéticos, Universidade do Porto, Campus Agrário de Vairão, Vairão, Portugal; ^3^Departamento de Microbiología, Facultad de Ciencias de la Salud, Universidad de Talca, Campus Talca, Talca, Chile; ^4^Centro de Ecología Integrativa, Universidad de Talca, Campus Talca, Talca, Chile; ^5^Serviço de Imunologia Básica e Clínica, Departamento de Patologia, Faculdade de Medicina da Universidade do Porto, Porto, Portugal; ^6^Centro de Investigação em Tecnologias e Serviços de Saúde (CINTESIS@RISE), Faculdade de Medicina da Universidade do Porto, Porto, Portugal; ^7^Serviço de Imunoalergologia, Centro Hospitalar Universitário São João (CHUSJ), Porto, Portugal; ^8^i3S—Instituto de Investigação e Inovação em Saúde, Universidade do Porto, Porto, Portugal; ^9^Ipatimup—Instituto de Patologia e Imunologia Molecular da Universidade do Porto, Porto, Portugal

**Keywords:** 16S rRNA, allergy, asthma, bacteriome, oral cavity, Portugal, rhinitis

## Abstract

Allergic rhinitis and asthma are two of the most common chronic respiratory diseases in developed countries and have become a major public health concern. Substantial evidence has suggested a strong link between respiratory allergy and upper airway dysbacteriosis, but the role of the oral bacteriota is still poorly understood. Here we used 16S rRNA massive parallel sequencing to characterize the oral bacteriome of 344 individuals with allergic rhinitis (AR), allergic rhinitis with asthma (ARAS), asthma (AS) and healthy controls (CT). Four of the most abundant (>2%) phyla (Actinobacteriota, Firmicutes, Fusobacteriota, and Proteobacteria) and 10 of the dominant genera (*Actinomyces, Fusobacterium, Gemella, Haemophilus, Leptotrichia, Neisseria, Porphyromonas, Prevotella, Streptococcus,* and *Veillonella*) in the oral cavity differed significantly (*p* ≤ 0.03) between AR, ARAS or AS and CT groups. The oral bacteriome of ARAS patients showed the highest intra-group diversity, while CT showed the lowest. All alpha-diversity indices of microbial richness and evenness varied significantly (*p* ≤ 0.022) in ARAS vs. CT and ARAS vs. AR, but they were not significantly different in AR vs. CT. All beta-diversity indices of microbial structure (Unifrac, Bray-Curtis, and Jaccard distances) differed significantly (*p* ≤ 0.049) between each respiratory disease group and controls. Bacteriomes of AR and ARAS patients showed 15 and 28 upregulated metabolic pathways (PICRUSt2) mainly related to degradation and biosynthesis (*p* < 0.05). A network analysis (SPIEC-EASI) of AR and ARAS bacteriomes depicted simpler webs of interactions among their members than those observed in the bacteriome of CT, suggesting chronic respiratory allergic diseases may disrupt bacterial connectivity in the oral cavity. This study, therefore, expands our understanding of the relationships between the oral bacteriome and allergy-related conditions. It demonstrates for the first time that the mouth harbors distinct bacteriotas during health and allergic rhinitis (with and without comorbid asthma) and identifies potential taxonomic and functional microbial biomarkers of chronic airway disease.

## 1. Introduction

Allergic rhinitis and asthma are two of the most common chronic airway diseases in developed countries, inflicting a severe health and economic burden to society (Meltzer and Bukstein, 2011; Serebrisky and Wiznia, 2019; Dierick et al., 2020). About 400 million people suffer from allergic rhinitis worldwide (Nur Husna et al., 2022), while over 300 million patients have been diagnosed with asthma (GBD Disease Injury Incidence Prevalence Collaborators, 2018; The Global Asthma Report, 2018; Dharmage et al., 2019; GBD Diseases Injuries Collaborators, 2020). Allergic rhinitis is an inflammation of the nasal membranes characterized by sneezing, congestion, itching, and rhinorrhea, in any combination (Steelant et al., 2016, 2018; Acevedo-Prado et al., 2022; Savoure et al., 2022), traditionally considered a disorder of the nose and nasal passages. However, emerging evidence suggests that it may represent a component of a systemic airway disease involving the entire respiratory tract (Small et al., 2018). Similarly, asthma is a multifactorial condition that also affects the entire airways and when triggered it leads to airway tightness, airway obstruction, inflammation and mucous production (Mims, 2015; Licari et al., 2018; Dharmage et al., 2019).

Allergic rhinitis and asthma frequently coexist (Compalati et al., 2010; Pite et al., 2014; Ferreira-Magalhaes et al., 2015; Small et al., 2018; Bousquet et al., 2019), which suggests that they may represent a combined airway inflammatory disease with several clinical, epidemiological and pathophysiological connections (Bergeron and Hamid, 2005; Pawankar, 2006; Kim et al., 2008; Compalati et al., 2010). They share, for example, the same profile of inflammation, mediators, adhesion molecules and some treatment approaches (Bergeron and Hamid, 2005). Moreover, rhinitis and asthma have an overlapping prevalence and similar health care and social costs in quality of life (Kim et al., 2008).

Considerable research has shown that the bacterial communities living in different sections of the upper and lower respiratory tract are connected and influence the onset and development of chronic airway disease (Dickson et al., 2013; Huang and Boushey, 2014; Castro-Nallar et al., 2015; Dickson and Huffnagle, 2015; Huang and Boushey, 2015; Pérez-Losada et al., 2015; Teo et al., 2015; Lynch, 2016; Perez-Losada et al., 2018; Losol et al., 2021; Chen et al., 2022; Pérez-Losada et al., 2023); but the role of the oral bacteriota has received much less attention (Zarco et al., 2012; Cai et al., 2022). The oral cavity harbors over 700 species of bacteria (Deo and Deshmukh, 2019), what makes it the second most diverse bacterial community in the human body after the gut (Zhang et al., 2018). Oral dysbacteriosis has been linked to multiple human diseases (e.g., dental caries, cancer, obesity, diabetes, Parkinson’s Disease), including also respiratory illnesses (e.g., pneumonia, chronic obstructive pulmonary disease, lung cancer, cystic fibrosis and asthma; Le Bars et al., 2017; Yamashita and Takeshita, 2017; Saeb et al., 2019; Dong et al., 2021; Pathak et al., 2021; Tuominen and Rautava, 2021; Zapala et al., 2022). The mouth is also the immune system’s first line of defense against most foreign antigens and some oral bacteria have been suggested to impact host immune and inflammatory responses (Abusleme et al., 2021; Cai et al., 2022). Furthermore, some emerging evidence is now hinted at a link between oral dysbacteriosis and chronic respiratory disease (Mammen et al., 2000; Dong et al., 2021; Pathak et al., 2021) and the emergence of opportunistic infections (Sedghizadeh et al., 2017; Dong et al., 2021).

Nonetheless, the host-bacteria interplay has only begun to be explored in allergy/asthma (Dzidic et al., 2018; Espuela-Ortiz et al., 2019; Durack et al., 2020; Cai et al., 2022; Pérez-Losada et al., 2023), while studies describing the oral bacteriota (commensal and pathogenic taxa) during allergic rhinitis are still missing. Hence, whether and how taxonomic and functional characteristics of the oral bacteriota may contribute to rhinitis and asthma remains poorly understood or unknown. Deciphering these relationships could ultimately improve our understanding of the pathophysiology of these two conditions alone or combined and help to identify new prognostic markers (Benton et al., 2010; Taylor et al., 2018).

To address this knowledge gap, we have coupled 16S rRNA massive parallel sequencing with metataxonomics to analyze 347 oral samples from individuals with allergic rhinitis (with and without comorbid asthma), asthma and healthy controls. We estimated individual taxonomic and functional profiles and inferred differences in microbial composition, diversity, metabolic pathways and microbe-microbe interactions between clinical groups.

## 2. Materials and methods

### 2.1. Cohort

ASMAPORT was a cross-sectional study of children and adults designed to find associations between airway microbes and clinical manifestations of rhinitis and asthma. Patients suspected to have allergic rhinitis or asthma attending the outpatient clinic of the Allergy and Clinical Immunology Department of the Centro Hospitalar Universitário São João in Porto (Portugal) were recruited between July 2018 and January 2020. Enrollment was performed at the first visit after completing a clinical history questionnaire, physical examination and allergy testing. Healthy volunteers from the Porto area with no history of respiratory illness were also enrolled, but did not complete the questionary or provided clinical information.

Allergic rhinitis diagnosis was confirmed by an allergy specialist based on clinical criteria (sneezing, nasal pruritus, rhinorrhea or congestion) and positive skin prick testing or specific IgE (ImmunoCAP, Thermo Fisher) to at least one common inhalant allergen (mites, pollens, molds, cat or dog dander; Pereira et al., 2006; Bousquet et al., 2009). Asthma diagnosis was established by the attending physician based on the presence of typical symptoms (wheeze, shortness of breath/chest tightness and cough) in the previous 12 months or a positive bronchodilator responsiveness test with salbutamol (FEV_1_ reversibility of at least 12% and 200 mL; Silva et al., 2019). No information on family history, auto-immune disorders, exposure to specific allergens, vaccination and professional occupation was collected for the participants.

### 2.2. Sampling

A total of 347 individuals participated in the study (Supplementary Table 1). They were distributed into four clinical groups: allergic rhinitis (AR = 53 individuals), allergic rhinitis with asthma (ARAS = 183), asthma (AS = 12) and healthy controls (CT = 99). Samples were collected by swabbing the buccal mucosa of the right and left cheeks for 30 s using the same cotton swab. Sample swabs were then preserved in tubes containing DNA/RNA Shield (Zymo Research) and stored at −20°C until further analysis.

### 2.3. 16S rRNA massive parallel sequencing

Total DNA was extracted from swabs using the ZymoBIOMICS™ DNA Miniprep Kit D4300. All extractions yielded > 2 ng/μ of total DNA, as indicated by NanoDrop 2000 UV–Vis Spectrophotometer measuring. DNA extractions were prepared for sequencing using the metataxonomic protocol (Marchesi and Ravel, 2015) described in Kozich et al. (2013). Each DNA sample was amplified for the V4 region (~250 bp) of the 16S rRNA gene and libraries were sequenced in a single run of the Illumina MiSeq sequencing platform at the University of Michigan Medical School. Negative controls processed as above showed no PCR band on an agarose gel. We used 10 water and reagent negative controls and seven mock communities (i.e., reference samples with a known composition) to detect contaminating microbial DNA within reagents and measure the sequencing error rate. We did not find evidence of contamination and our sequencing error rate was as low as 0.0054%. Sequence files and associated metadata and BioSample attributes for all samples used in this study have been deposited in the NCBI (PRJNA913468). Metadata and ASV abundances with corresponding taxonomic classifications are presented in Supplementary Tables 1, 2, respectively.

### 2.4. Metataxonomic analyses

16S rRNA–V4 amplicon sequence variants (ASV) in each sample were inferred using the DADA2 version 1.18 (Callahan et al., 2016). Reads were filtered using standard parameters, with no uncalled bases, maximum of 2 expected errors and truncating reads at a quality score of 2. Forward and reverse reads were trimmed after 150 bases, merged and chimeras were identified. Taxonomic assignment was performed against the SILVA v138.1 database using the RDP naive Bayesian classifier (Wang et al., 2007; Quast et al., 2013). ASV sequences (~250 bp) were aligned in MAFFT (Katoh and Standley, 2013) and used to build a tree with FastTree (Price et al., 2010). The resulting ASV tables and phylogenetic tree were imported into phyloseq (McMurdie and Holmes, 2013) for further analysis. We normalized our samples using the negative binomial distribution as recommended by McMurdie and Holmes (2014) and implemented in the Bioconductor package DESeq2 (Love et al., 2014). This approach simultaneously accounts for library size differences and biological variability and has increased sensitivity if groups include less than 20 samples (Weiss et al., 2017). Taxonomic and phylogenetic alpha-diversity were estimated using Chao1 richness and Shannon, ACE, and Phylogenetic (Faith’s) diversity indices. Beta-diversity was estimated using phylogenetic Unifrac (unweighted and weighted), Bray-Curtis and Jaccard distances, and dissimilarity between samples was explored using principal coordinates analysis (PCoA). Because of the sample size of the AS group, we only used it in some of the pairwise comparisons and applied statistical tests that are moderately robust to small sample sizes (Hilty et al., 2010; Castro-Nallar et al., 2015; Pérez-Losada et al., 2015; Lal et al., 2017; Fazlollahi et al., 2018).

Differences in taxonomic composition (phyla and genera) and alpha-diversity indices between respiratory disease groups (AR, ARAS and AS) and healthy individuals (CT) were assessed using the Wilcoxon and the Kruskal-Wallis rank sum tests and the Wald test with Cook’s distance correction for outliers (DESeq2 package), while accounting for covariables (age, season and sex). Beta-diversity indices were compared using permutational multivariate analysis of variance (adonis) as implemented in the Vegan R package (Dixon, 2003), while also accounting for covariables. None of the covariables resulted significant for any of the taxonomic and diversity indices compared. We applied the Benjamini-Hochberg method at alpha = 0.05 to correct for multiple hypotheses testing (Cook, 1977; Benjamini and Hochberg, 1995). All the analyses were performed in R (R Core Team, 2008) and RStudio (RStudio, 2015). A full record of all statistical analyses was created in RStudio and is included in Supplementary Figure 1. All data files and R code used in this study with instructions can be found in GitHub: https://github.com/mlosada323/asmaport_bacteriome_oral.

### 2.5. Functional prediction

Bacterial function was predicted by coupling the Phylogenetic Investigation of Communities by Reconstruction of Unobserved States (PICRUSt2) software (Douglas et al., 2020) with the Integrated Microbial Genomes and Microbiomes (IMG/M) database (Chen et al., 2021). ASVs abundances were normalized by 16S rRNA gene copy number and gene abundances were estimated by multiplying the normalized ASV counts by the predicted gene copy numbers. Metabolic pathways were predicted in the MetaCyc database (Caspi et al., 2018, 2020) using gene abundance predictions and PICRUSt2 default parameters. Differentially abundant metabolic pathways were analyzed using the DESEq2 Wald’s test with a value of *p* cutoff of 0.05 and an a log2FC of 2. The AS group was excluded due to its inadequate sample size for this analysis.

### 2.6. Network analyses

Community interactions among bacterial taxa were inferred using the network approach implemented in the SPIEC-EASI (SParse InversE Covariance Estimation for Ecological Association Inference) R package (Kurtz et al., 2015). All ASVs were classified to their best-hit taxonomic assignment and agglomerated by identical taxonomic rank. The most parsimonious network structures were detected using LASSO regularized regression by calling the neighborhood selection method (method = “mb”) on the inverse covariance matrix. In order to capture the optimal network links, optimal lambda values were chosen by nlambda = 50 and lambda.min.ratio = 0.01 using 50 subsamples for graph re-estimation (rep.num = 50). Count data were centered log-ratio transformed. The number of links per node was chosen as the centrality metric to detect hub nodes (Degree centrality metric). The clustering/modulation stage was performed with the default method for the association matrices in the NetCoMi R package (Peschel et al., 2021). All nodes whose normalized degree centrality metric was >90 percentile were defined as hub nodes. Finally, network visualizations were generated using the NetCoMi plot function. The small AS group was also excluded from this analysis.

## 3. Results

We studied a cohort of 347 participants (248 individuals with respiratory disease and 99 healthy controls) comprised mainly of children and young adults (Supplementary Table 1). The age of the participants varied from 4 to 58 years (mean = 12.6 ± 5.2 years) and 47.5:52.5% were male:female. We sequenced the variable V4 region of the 16S rRNA gene (~250 bp) to characterize the oral bacteriome of each participant. ASV singletons, two CT samples and one ARAS sample with <7,114 reads were eliminated, rendering a final number of 344 individual samples.

### 3.1. Taxonomic diversity and microbial structure

The oral bacteriome of the 344 samples analyzed after quality control included 7,455,683 reads, ranging from 7,114 to 68,288 sequences per sample (mean = 21,674), and comprised 2,172 ASVs (Supplementary Table 2). AR samples had 180 unique ASVs, ARAS samples had 806, AS samples had 36 and CT samples had 294. The four groups shared 338 ASVs, while other pairs and trios shared a variable number of ASVs (0 to 161 ASVs; Supplementary Figure 2).

**Figure 1:**
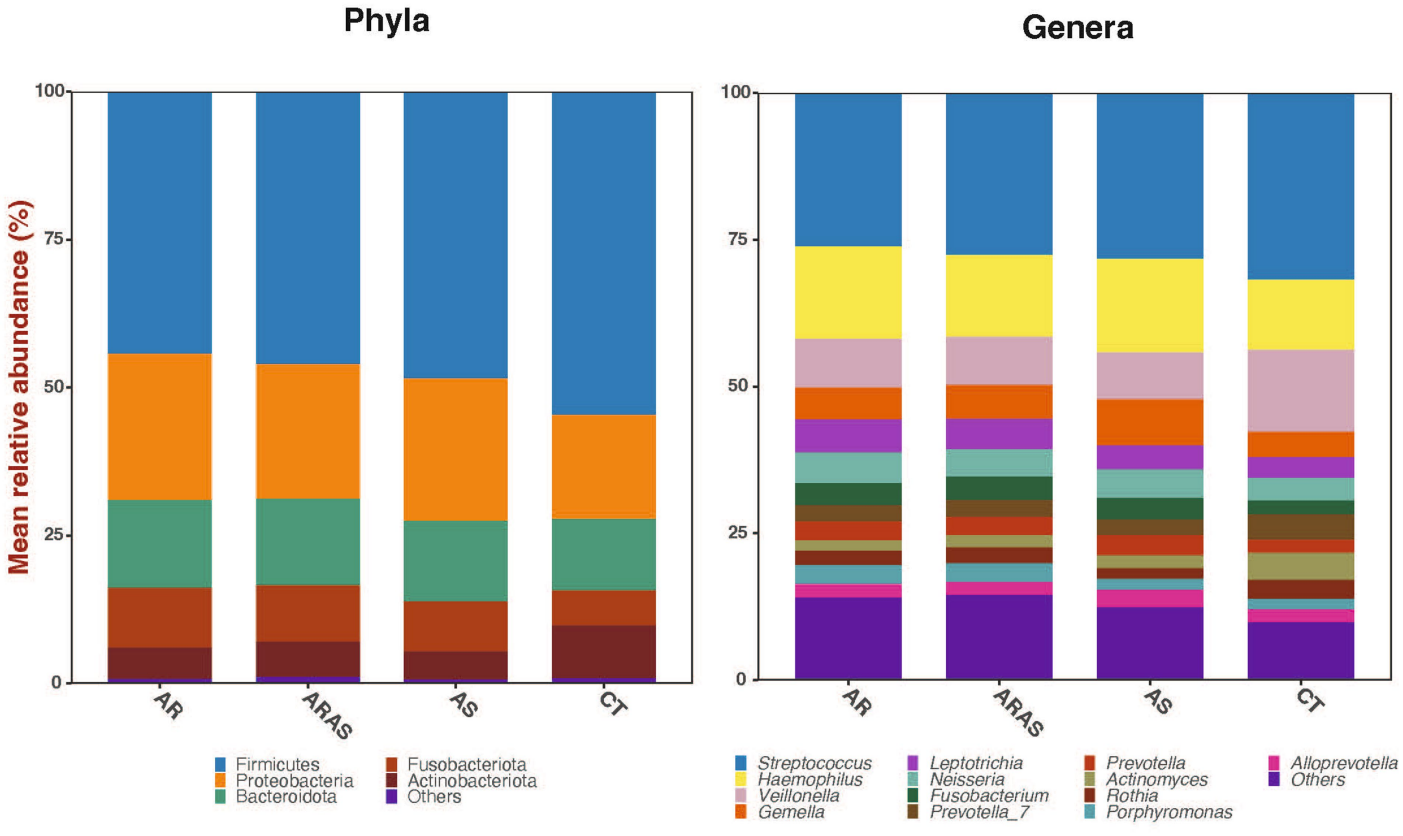
Bar plots of mean relative proportions of the top bacterial phyla and genera in the oral cavity of participants with allergic rhinitis (AR), AR with comorbid asthma (ARAS), asthma (AS), and healthy controls (CT).

The bacterial sequences across all 344 filtered samples were classified into five dominant (>2% abundance) Phyla: Firmicutes (49.3%), Proteobacteria (21.1%), Bacteroidota (13.8%), Fusobacteriota (8.2%) and Actinobacteriota (6.7%; Figure 1). Those Phyla consisted of 13 dominant (>2%) genera: *Streptococcus* (29.3%), *Haemophilus* (13.2%), *Veillonella* (10.1%), *Gemella* (5.4%), *Leptotrichia* (4.6%), *Neisseria* (4.6%), *Prevotella*_7 (3.3%), *Fusobacterium* (3.3%), *Actinomyces* (2.9%), *Prevotella* (2.8%), *Rothia* (2.7%), *Porphyromonas* (2.7%) and *Alloprevotella* (2.2%; Figure 1).

Twelve ASVs (ASV69, ASV24, ASV5, ASV9, ASV15, ASV33, ASV14, ASV11, ASV25, ASV1, ASV35, and ASV8) of the genera *Corynebacterium*, *Actinomyces*, *Haemophilus*, *Neisseria*, *Lautropia*, *Veillonella*, *Streptococcus*, *Granulicatella* and *Gemella* comprised the oral core bacteriome of respiratory disease patients and accounted for 52.2% of the total reads. Seven ASVs (ASV5, ASV14, ASV11, ASV25, ASV1, ASV35, and ASV8) of the genera *Haemophilus*, *Veillonella*, *Streptococcus*, *Granulicatella* and *Gemella* comprised the oral core bacteriome of healthy patients and accounted for 42.3% of the total reads. These core ASVs may represent the more stable and consistent bacterial members of the oral cavity (Backhed et al., 2012; Shade and Handelsman, 2012).

Two to four of the dominant phyla (Figure 1) showed significant differences (*p* ≤ 0.03; Wilcoxon test) in their mean relative proportions between AR, ARAS or AS and CT (Table 1). Similarly, three to nine dominant genera (Figure 1) showed significant differences in their mean relative proportions between AR, ARAS or AS and CT (Table 1). These results were confirmed by the Wald test with Cook’ s distance correction for outliers (0.03 ≤ *p* ≤ 0.0001). None of the dominant phyla or genera varied significantly between respiratory disease groups.

**Table 1:** Mean relative proportions (%) of bacterial phyla and genera in the oral bacteriome of participants with allergic rhinitis (AR), AR with comorbid asthma (ARAS), asthma (AS) and healthy controls (CT).

	Mean relative proportions (%)				Wilcoxon test significance		
	AR	ARAS	AS	CT	AR-CT	ARAS-CT	AS-CT
**Phylum**							
Actinobacteriota	5.5	5.8	4.5	8.6	<0.001	<0.001	<0.001
Bacteroidota	15.3	14.8	13.0	11.9	ns	ns	ns
Firmicutes	43.6	46.1	50.3	55.9	0.009	0.002	ns
Fusobacteriota	10.3	9.4	8.0	5.8	<0.001	<0.001	0.03
Proteobacteria	24.5	22.8	23.7	17.1	0.009	ns	ns
**Genus**							
*Actinomyces*	1.9	2.1	2.2	4.5	<0.001	<0.001	<0.001
*Alloprevotella*	2.4	2.2	3.2	2.2	ns	ns	ns
*Fusobacterium*	3.9	3.9	3.4	2.2	<0.001	<0.001	0.03
*Gemella*	5.3	5.8	9.0	4.4	ns	0.001	ns
*Haemophilus*	15.3	13.7	15.9	11.5	0.009	ns	ns
*Leptotrichia*	5.9	5.2	3.7	3.5	<0.001	<0.001	ns
*Neisseria*	5.3	5.0	4.6	3.8	0.002	0.004	ns
*Porphyromonas*	3.1	3.2	1.9	1.8	<0.001	<0.001	ns
*Prevotella*	3.3	3.1	3.1	2.1	0.009	<0.001	ns
*Prevotella_7*	2.9	3.0	2.2	4.2	ns	ns	ns
*Rothia*	2.5	2.5	1.6	3.2	ns	ns	ns
*Streptococcus*	25.5	27.6	29.1	33.2	0.009	0.002	ns
*Veillonella*	8.4	8.1	7.6	13.8	<0.001	<0.001	0.017

*p*-values for significant pairwise comparisons (Wilcoxon test) are also displayed. ns, not significant.

**Figure 2:**
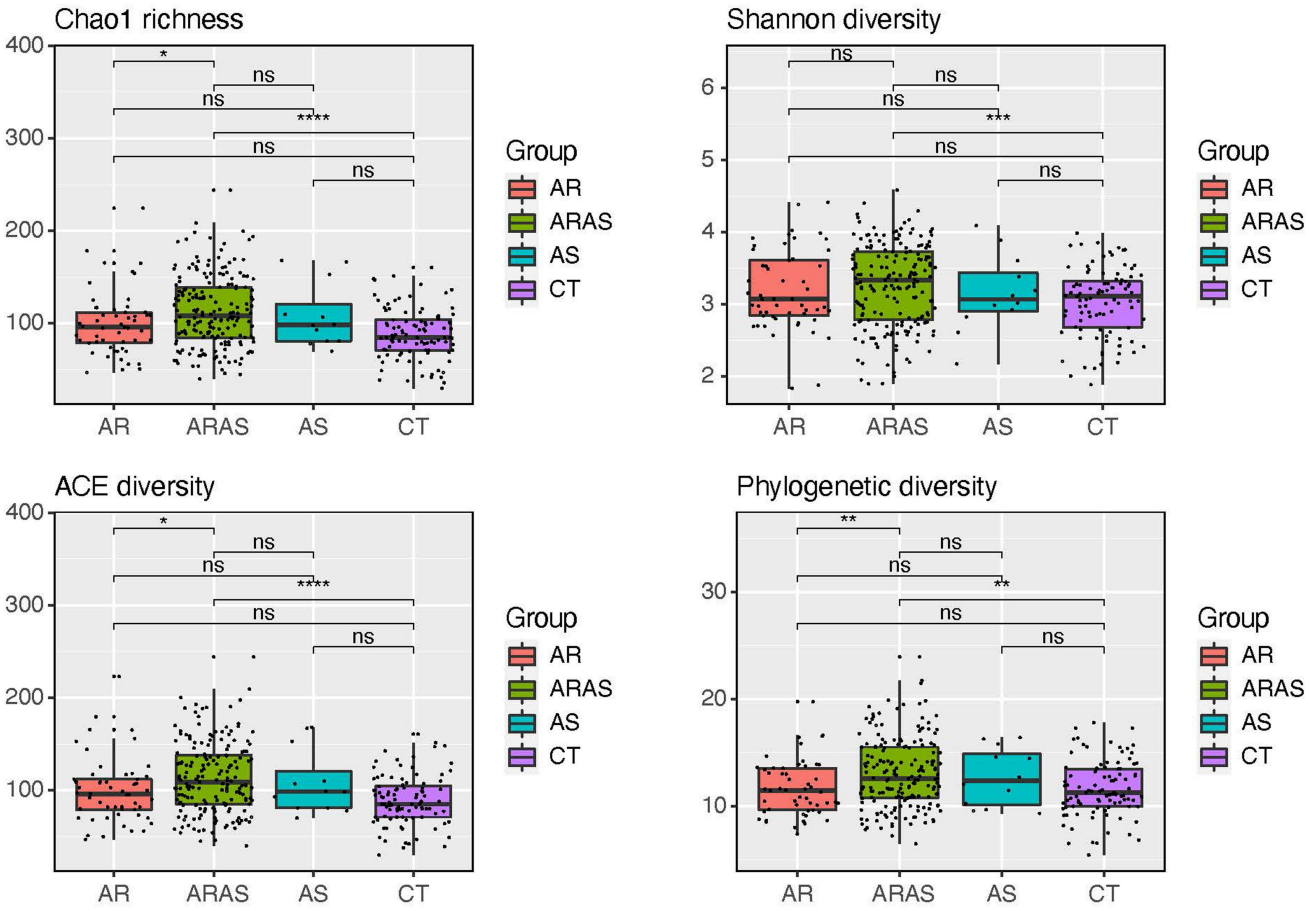
Alpha-diversity estimates (Chao1, Shannon, ACE, and phylogenetic diversity) of bacterial diversity and statistical significance (Wilcoxon test) in participants with allergic rhinitis (AR), AR with comorbid asthma (ARAS), asthma (AS) and healthy controls (CT). ns = not significant; ^*^p ≤ 0.05; ^**^p ≤ 0.01; ^***^p ≤ 0.001; ^****^p ≤ 0.0001.

Alpha-diversity indices (Shannon, Chao1, ACE, and PD) of microbial community richness and evenness varied among clinical groups (Figure 2; Supplementary Table 3). ARAS showed the highest diversity for all indices, while CT showed the lowest. ARAS vs. CT was significant for all diversity indices (Wilcoxon test; *p* ≤ 0.0012), while ARAS vs. AR resulted significant for three of them (Wilcoxon test; *p* ≤ 0.022). All the other pairwise comparisons were not significant.

**Figure 3:**
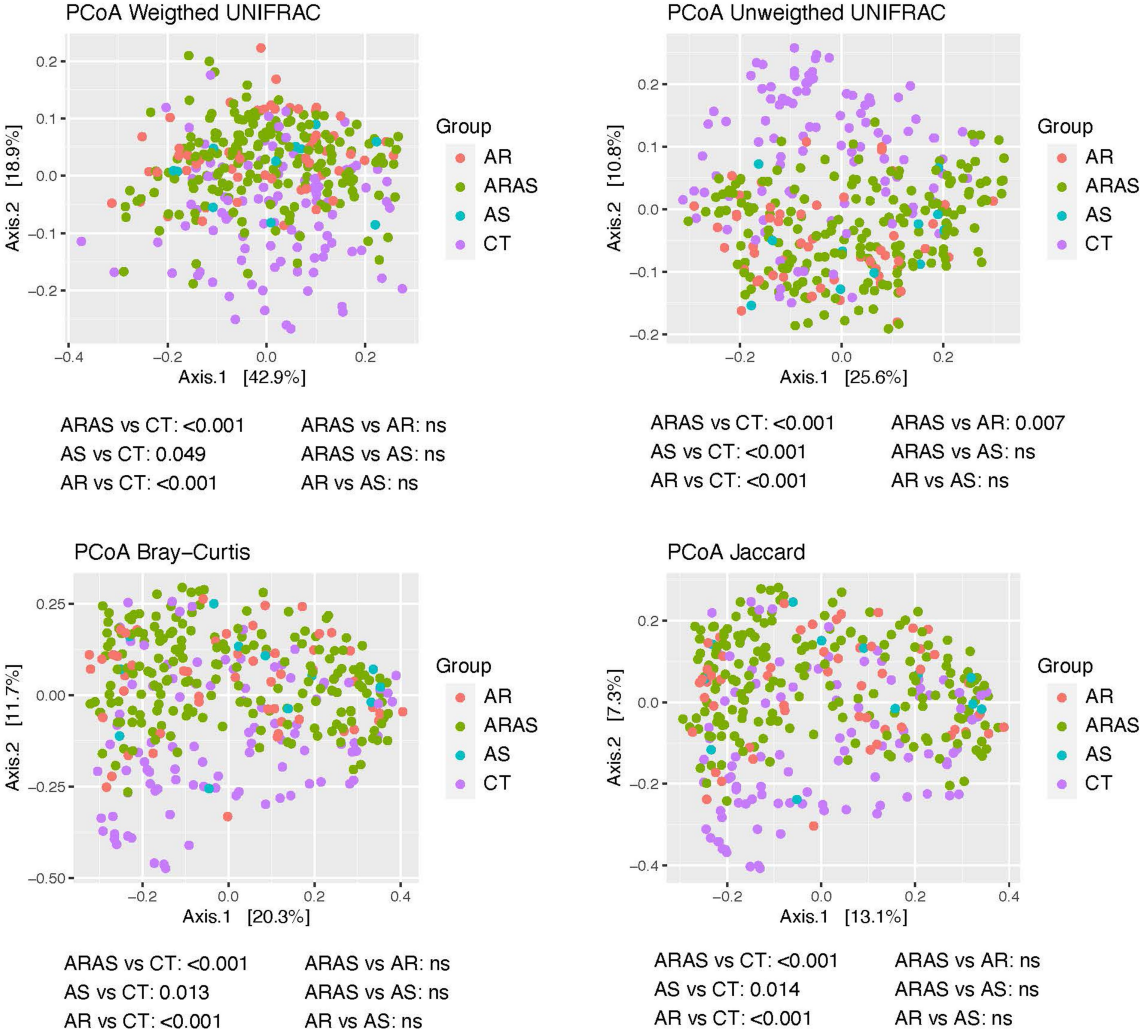
Principal coordinates analysis (PCoA) plots of beta-diversity estimates (Unifrac, Bray-Curtis and Jaccard indices) and statistical significance (adonis test) in the oral bacteriomes of participants with allergic rhinitis (AR), AR with comorbid asthma (ARAS), asthma (AS) and healthy controls (CT). ns, not significant.

Our PCoAs (Figure 3) of beta-diversity estimates showed partial segregation of the oral bacteriotas from each clinical group for all distances (Unifrac, Bray-Curtis and Jaccard). The adonis analyses detected significant differences (*p* ≤ 0.049) in community structure (beta-diversity) between AR, ARAS or AS and CT for all distances. Additionally, ARAS vs. AR also resulted significant (*p* = 0.007) for the unweighted Unifrac distance (Figure 3). None of the other pairwise comparisons showed significant differences in beta-diversity. This suggests that the oral bacteriomes of AR, ARAS, and AS participants may differ from those of healthy individuals in a similar compositional manner.

### 3.2. Functional diversity

We predicted (PICRUSt2) bacterial functional profiles for the AR, ARAS and CT groups (Supplementary Table 4) and then compared AR and ARAS against CT subjects to infer differentially abundant pathways (Supplementary Figure 3). We detected 15 upregulated pathways in AR vs. CT and 28 upregulated pathways in ARAS vs. CT, but only eight upregulated pathways in ARAS vs. AR. The 15 pathways detected in AR vs. CT were also detected in ARAS vs. CT; thus, suggesting again that bacteriomes of AR and ARAS participants may deviate from those of healthy individuals in a similar manner. Most of the pathways in all comparisons were related to degradation and biosynthesis processes.

### 3.3. Network interactions

**Figure 4:**
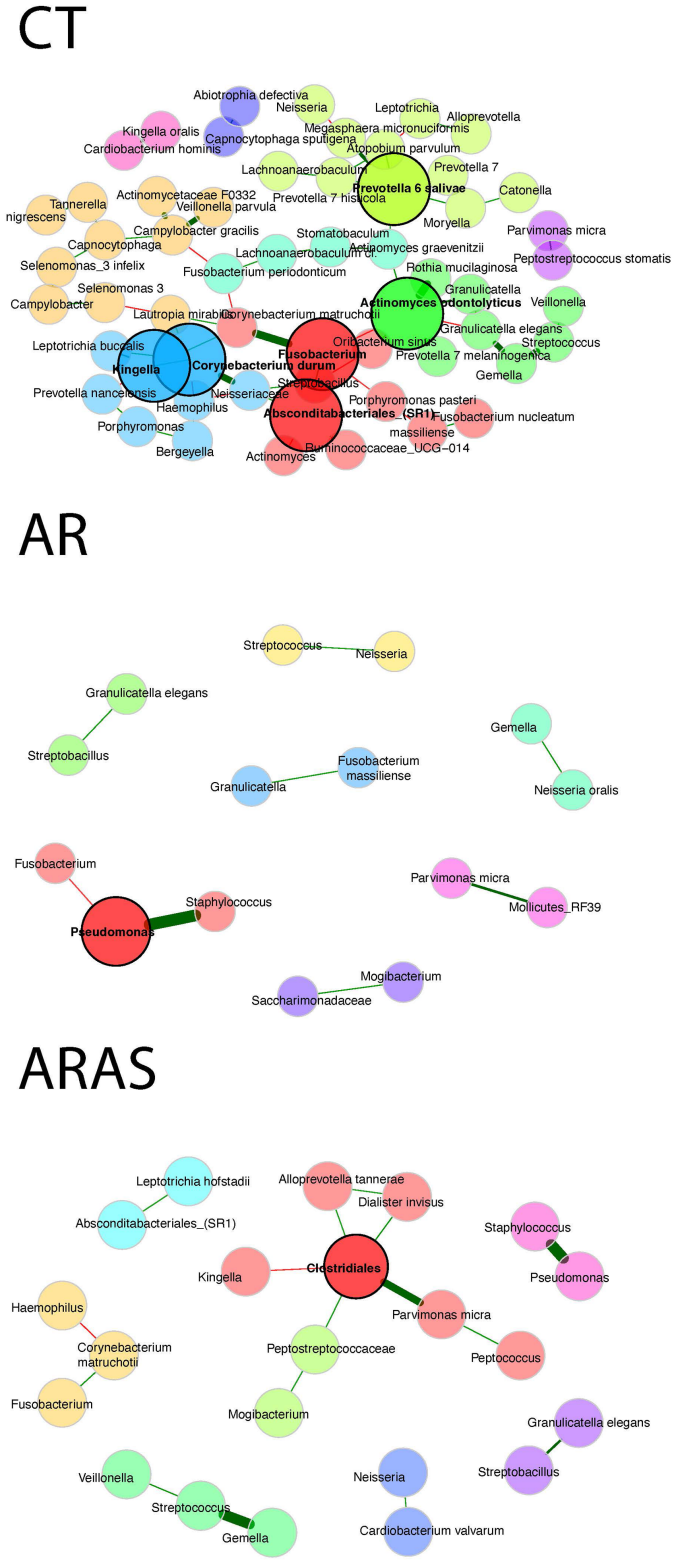
Co-occurrence networks of bacterial taxa in the oral bacteriomes of participants with allergic rhinitis (AR), AR with comorbid asthma (ARAS) and healthy controls (CT). Nodes represent taxa connected by edges whose width is proportional to the strength of their association. Green and red edges indicate positive and negative correlations, respectively. Nodes within the 90th percentile of degree connectivity are considered hub nodes (black circle line). Inferred modules or subnetworks are colored differently.

We inferred interactions among bacteria in the AR, ARAS, and CT groups using SPIEC-EASI networks (Figure 4). The bacterial network of the control group was more complex than those of the AR and ARAS groups. The CT network included nine modules (subnetworks), six hubs or key taxa (*Kingella*, *Corynebacterium durum*, Absconditabacteriales_(SR1), *Fusobacterium*, *Prevotella* 6 *salivae*, and *Actinomyces odontolyticus*), 72 nodes and 57 connected nodes. The AR network included seven modules, one hub taxa (*Pseudomonas*), 43 nodes and 15 connected nodes. The ARAS network included eight modules, one hub taxa (*Clostridiales*), 47 nodes and 22 connected nodes. No hub taxa were shared among clinical groups. All of the 13 dominant genera in the oral bacteriome (Table 1) were assembled into subnetworks in the CT group, while eight genera were assembled in the ARAS group and four genera in the AR group. Some common genera of the upper airways including pathogenic species (e.g., *Streptococcus, Staphylococcus, Neisseria, Haemophilus* and *Pseudomonas*) formed subnetworks with other commensal and pathogenic genera. Interestingly, *Streptococcus* and *Neisseria* or *Pseudomonas* and *Staphylococcus* were only connected in the ARAS or AR groups alone or associated to *Fusobacterium*, which also includes several periodontal pathogens and other opportunistic species associated to oral infections (Szafranski et al., 2015).

## 4. Discussion

Allergic rhinitis and asthma are common chronic respiratory diseases that cause major distress worldwide (Meltzer and Bukstein, 2011; Serebrisky and Wiznia, 2019; Dierick et al., 2020). Significant progress has been made toward understanding the role of the airway bacteriota in respiratory disease, but the impact of oral dysbacteriosis in allergic rhinitis and asthma is still understudied (Zarco et al., 2012; Dong et al., 2021; Cai et al., 2022). Here we present the results of a cross-sectional study comparing the oral bacteriotas of 344 individuals with allergic rhinitis (with and without comorbid asthma), asthma and healthy controls.

The oral bacteriomes were composed of five dominant phyla and 13 dominant genera (Figure 1; Table 1). All these taxa have been previously described in the oral cavity of healthy individuals, where they are common residents (Sedghi et al., 2000; Yamashita and Takeshita, 2017; Escapa et al., 2018; Pathak et al., 2021; Bourgeois et al., 2022; Varoni and Rimondini, 2022). Our oral samples consisted mainly of commensal genera (e.g., *Actinomyces, Fusobacterium, Leptotrichia, Porphyromonas, Prevotella* and *Streptococcus*; Avila et al., 2009; Escapa et al., 2018), but some genera including pathogenic species previously associated to asthma and allergy in both the mouth (Dzidic et al., 2018; Espuela-Ortiz et al., 2019; Durack et al., 2020) and nose (Castro-Nallar et al., 2015; Pérez-Losada et al., 2015; Losol et al., 2021; Chiang et al., 2022; Pérez-Losada et al., 2023) were also detected in patients with rhinitis. Hence, our results support the potential role of the mouth as a reservoir for opportunistic respiratory pathogens (Dong et al., 2021), which can then the enrich nasal–pharyngeal bacteriota (Fan et al., 2020).

Healthy participants and those with chronic respiratory illnesses bear unique microbial taxa in their oral cavity. The oral bacteriome from healthy controls contained 13.5% unique ASVs, while the AR, ARAS, and AS bacteriomes contained 8.3%, 37.1%, and 1.7% unique ASVs, respectively (Supplementary Table 2; Supplementary Figure 2). These ASVs are potential biomarkers of disease for each group. Future metataxonomic and metagenomic studies will need to confirm their consistency over time and across oral microenvironments, and their potential as therapeutic targets for rhinitis and asthma (Castro-Nallar et al., 2015; Pérez-Losada et al., 2015; Chung, 2017).

The mean relative proportions of up to 80% and 70% of the dominant oral bacterial phyla and genera, respectively, varied significantly between healthy samples and respiratory disease groups (Table 1). The most striking differences were observed between AR or ARAS and CT, where three or four phyla and nine genera varied in their mean relative abundances, respectively. Fusobacteriota and Proteobacteria were significantly more abundant in AR or ARAS, while Actinobacteriota and Firmicutes were significantly more abundant in CT. Similarly, *Fusobacterium*, *Gemella, Haemophilus, Leptotrichia, Neisseria, Porphyromonas* and *Prevotella* were significantly more abundant in AR or ARAS, while *Actinomyces, Streptococcus* and *Veillonella* were significantly more abundant in healthy subjects. No other studies so far have characterized the oral bacteriota of patients with rhinitis.

Two phyla and three genera varied significantly between AS and CT, despite the small sample size of this group. Fusobacteriota was significantly more abundant in the asthmatic group, while Actinobacteriota was significantly more abundant in CT. Similarly, *Fusobacterium* was more abundant in AS, while *Actinomyces* and *Veillonella* were more abundant in healthy subjects. Only three other studies have characterized the oral bacteriota of asthmatic children or adults (Dzidic et al., 2018; Espuela-Ortiz et al., 2019; Durack et al., 2020); they have revealed the same trend as here for *Actinomyces*, the opposite for *Fusobacterium* and discordant results for *Veillonella*. Compositional changes in these bacterial groups may provide insights into the pathobiology of allergic rhinitis and asthma. New cross-sectional and longitudinal studies using more powerful technologies (i.e., metagenomics) are required to validate our results and untangle the relationship between microbial colonization, dysbacteriosis and chronic inflammatory disease (Zarco et al., 2012; Lal et al., 2017; Cai et al., 2022; Losol et al., 2022).

Bacterial alpha-diversity (species richness and evenness) only varied significantly between ARAS and CT and between ARAS and AR (Figure 2). No studies so far have explored the diversity of the oral bacteriota in individuals with rhinitis, but a few studies have compared bacterial diversity and richness in children and adults with allergy or asthma. Espuela-Ortiz et al. (2019) showed that asthmatic children and adults have significantly higher diversity in the salivary microbiome than controls, while Durack et al. (2020) showed no differences between similar groups in adults. Dzidic et al. (2018), however, showed that children age seven with allergic diseases, particularly asthma, had significantly lower salivary bacterial diversity when compared to healthy children. Alpha-diversity has also shown inconsistent patterns in other studies of the upper airway bacteriome. Some studies of the nasal bacteriota, for example, have shown lower alpha-diversity rates in healthy controls compared to participants with asthma (Huang et al., 2011; Fazlollahi et al., 2018; Espuela-Ortiz et al., 2019) and allergic rhinitis, with and without comorbid asthma (Choi et al., 2014; Gan et al., 2021; Kim et al., 2022); while others have depicted the opposite trend across those same groups (Depner et al., 2017; Lal et al., 2017; Chen et al., 2022; Chiang et al., 2022; Losol et al., 2022) or among metrics of richness and evenness (Castro-Nallar et al., 2015; Chiang et al., 2022). A recent study (Chen et al., 2022) has also suggested that asthma may affect alpha-diversity in the upper airways more than allergic rhinitis. Our study seems to confirm that observation for the oral microbiota (Supplementary Table 3; Figure 2), since patients with AR and comorbid asthma (ARAS) had higher intra-sample diversity than those with just AR or CT. Nonetheless, given the discrepancy in alpha-diversity patterns in both the oral and nasal cavities, within sample diversity may not reliably capture disease status or pathogenesis in the upper airways.

AR, ARAS and AS samples displayed significant differences in community structure (i.e., beta-diversity) compared to those of healthy controls (Figure 3). This pattern held for all the distance metrics used, whether accounting for phylogenetic diversity or not. No differences were observed between AR and ARAS groups except for the unweighted Unifrac distance (Figure 3). Previous studies on the oral bacteriota in asthma or allergy were inconclusive or showed differences in structural patterns between healthy and allergic/asthmatic children 7 years old (Dzidic et al., 2018; Durack et al., 2020). However, multiple studies of the nasal bacteriota have showed specific community structuring associated with distinct bacterial composition in AR, ARAS or AS vs. healthy controls (Castro-Nallar et al., 2015; Depner et al., 2017; Lal et al., 2017; Fazlollahi et al., 2018; Bender et al., 2020; Chen et al., 2022; Chiang et al., 2022; Kim et al., 2022; Pérez-Losada et al., 2023). Hence, all these results combined suggest that bacterial compositional shifts may be a reliable predictor of disease status in the upper airways, given their lower stochasticity associated to dysbiosis (Ma et al., 2019; Ma, 2020; Pérez-Losada et al., 2023).

Airway microbiota can impact epithelial cell growth and repair and host inflammatory and immune responses, therefore affecting respiratory disease onset and progression (Costello et al., 2012; Castro-Nallar et al., 2015; Pérez-Losada et al., 2015; Lynch, 2016; Hoffman et al., 2017; Huffnagle et al., 2017; Ta et al., 2018; Lira-Lucio et al., 2020; Paudel et al., 2020; Losol et al., 2021; Chiang et al., 2022). Our PICRUSt2 analyses predicted 15 and 28 pathways upregulated in the oral microbiome of AR and ARAS patients, respectively, compared to CT participants (Supplementary Figure 3). No other studies so far have applied this approach to predict metabolic functions in the oral microbiome of asthmatic or rhinitic patients. However, previous research in other sections of the airways (Pérez-Losada et al., 2015; Li et al., 2019; Al Bataineh et al., 2020; Chiu et al., 2020; Hu et al., 2020; Samra et al., 2021; Chiang et al., 2022; Pérez-Losada et al., 2023) has suggested that some of the microbial metabolic pathways predicted here involved in amino acid (e.g., pyrimidine, leucine and arginine) and carbohydrate (e.g., peptidoglycan complexes and mannan) biosynthesis and degradation and metabolism (e.g., glycolysis and TCA cycle) may be associated with allergic sensitization, IgE sensitivity and inflammation of the airways and host immune response (e.g., superpathway of UDP-N-acetylglucosamine-derived O-antigen building blocks biosynthesis; Caspi et al., 2020; Chiu et al., 2021). Thus, our study suggests that oral dysbacteriosis may alter bacterial community functionality, thereby affecting the occurrence of allergic rhinitis or asthma. Nonetheless, since PICRUSt2 only predicts microbial function based on 16S rRNA reads, more powerful dual-transcriptomic approaches (e.g., Castro-Nallar et al., 2015; Pérez-Losada et al., 2015) should be applied in a longitudinal study to confirm these results.

Our co-occurrence network analyses depicted distinct and specific connectivity patterns of interactions in AR and ARAS groups compared to healthy controls (Figure 4). Each group network represents a different set of co-regulated bacteria that, in turn, suggests a distinct community partition (Sharma et al., 2019). The AR and ARAS networks were remarkably simpler than that of the control group, which contained more and larger modules and connected taxa. The most abundant and resident genera of the oral microbiota were embedded in the CT network, with some commensal taxa displaying numerous connections (e.g., *Actinomyces, Fusobacterium*, and *Prevotella*). Interestingly, these same genera did not form complex subnetworks in the AR and ARAS, which tended to be comprised of smaller clusters of pathogenic genera (e.g., *Streptococcus, Staphylococcus, Neisseria, Haemophilus*, and *Pseudomonas*). Microbe-microbe oral interactions have not been previously investigated in rhinitis; however, a few studies comparing asthmatic groups and healthy controls have revealed the same trends in connectivity observed here (Perez-Losada et al., 2018; Sharma et al., 2019; Huang et al., 2020; Kim et al., 2021). Altogether, these results suggest that “healthy” oral bacteriotas establish more complex relationship between their members than “diseased” bacteriotas, despite having lower bacterial diversity. Chronic airway disease seems to disrupt patterns of connectivity in the oral microbiota. Nonetheless, data are still incipient; further research is needed to characterize microbial networks and understand their role in allergic airway pathogenesis (Sanders et al., 2021).

The oral bacteriomes of patients with AR and ARAS showed partial differences in composition (Figure 2), structure (Figure 3) and metabolism (Supplementary Figure 3), but not in taxa abundances. Patterns of bacterial co-occurrence also varied between these two groups (Figure 4). A previous study (Chen et al., 2022) also described differences in intra-and inter-sample diversity between AR and ARAS bacteriomes in the nasal cavity, but not in their taxon proportions. It has been proposed that the association between allergic rhinitis and asthma may enhance lower respiratory tract inflammation (Oka et al., 2014; Chen et al., 2022), which may in turn lead to shifts in the oral bacteriotas of AR and ARAS groups.

## 5. Conclusion

The oral cavity is the first site of encounter between a majority of foreign antigens and the immune system; hence one could expect that the oral bacteriota will impact the onset and development of allergy-related diseases. We characterized for the first time the bacteriomes of the buccal mucosa in children and young adults with allergic rhinitis, with and without comorbid asthma, and compared them to healthy controls. Our analyses demonstrated that the oral bacteriotas of these groups harbor unique taxa (i.e., potential biomarkers of disease), are compositionally and structurally distinct, encode different metabolic functions and establish different microbe-microbe connections among their members. We show that oral dysbacteriosis may contribute to chronic allergic disease and warrants further study to better understand the relationship between the oral bacteriome and airway pathology.

## Data availability statement

The original contributions presented in the study are publicly available. The data presented in the study are deposited in the NCBI repository, accession number PRJNA913468.

## Ethics statement

The studies involving human participants were reviewed and approved by Comissão de Ética para a Saúde of the Centro Hospitalar Universitário São João/Faculdade de Medicina (Porto) March 2017—Parecer_58-17. Written informed consent to participate in this study was provided by the participants’ legal guardian/next of kin.

## Author contributions

MP-L, MO, and LD designed the experiment. MO and MP-L collected the samples. JL, TA, and LD collected and interpreted the clinical data. MP-L, EC-N, and VB-F analyzed the data. MP-L wrote the first draft of the manuscript. All authors contributed to the article and approved the submitted version.

## Funding

This study was co-funded by the EU via European Regional Development Fund (ERDF) and by national funds via the Fundação para a Ciência e a Tecnologia (FCT) and the project PTDC/ASP-PES/27953/2017—POCI-01-0145-FEDER-027953. MP-L was supported by the FCT under the “Programa Operacional Potencial Humano—Quadro de Referência Estratégico” Nacional funds from the European Social Fund and Portuguese “Ministério da Educação e Ciência” IF/00764/2013.

## Conflict of interest

The authors declare that the research was conducted in the absence of any commercial or financial relationships that could be construed as a potential conflict of interest.

## Publisher’s note

All claims expressed in this article are solely those of the authors and do not necessarily represent those of their affiliated organizations, or those of the publisher, the editors and the reviewers. Any product that may be evaluated in this article, or claim that may be made by its manufacturer, is not guaranteed or endorsed by the publisher.

## Supplementary material

SUPPLEMENTARY FIGURE 1 Full account of statistical analysis performed in R.

SUPPLEMENTARY FIGURE 2 UpSet plots of amplicon sequence variants (ASVs) in the oral bacteriomes of participants with allergic rhinitis (AR), AR with comorbid asthma (ARAS), asthma (AS) and healthy controls (CT).

SUPPLEMENTARY FIGURE 3 Differential abundance analysis (Wald’s test) of functional profiles in the oral bacteriomes of participants with allergic rhinitis (AR), AR with comorbid asthma (ARAS) and healthy controls (CT).
