# Supplementary figures and images for: The oral bacteriomes of patients with allergic rhinitis and asthma differ from that of healthy controls
Source: Front Microbiol. 2023 Jun 7;14:1197135. doi: 10.3389/fmicb.2023.1197135 (PMC10335798; doi:10.3389/fmicb.2023.1197135)

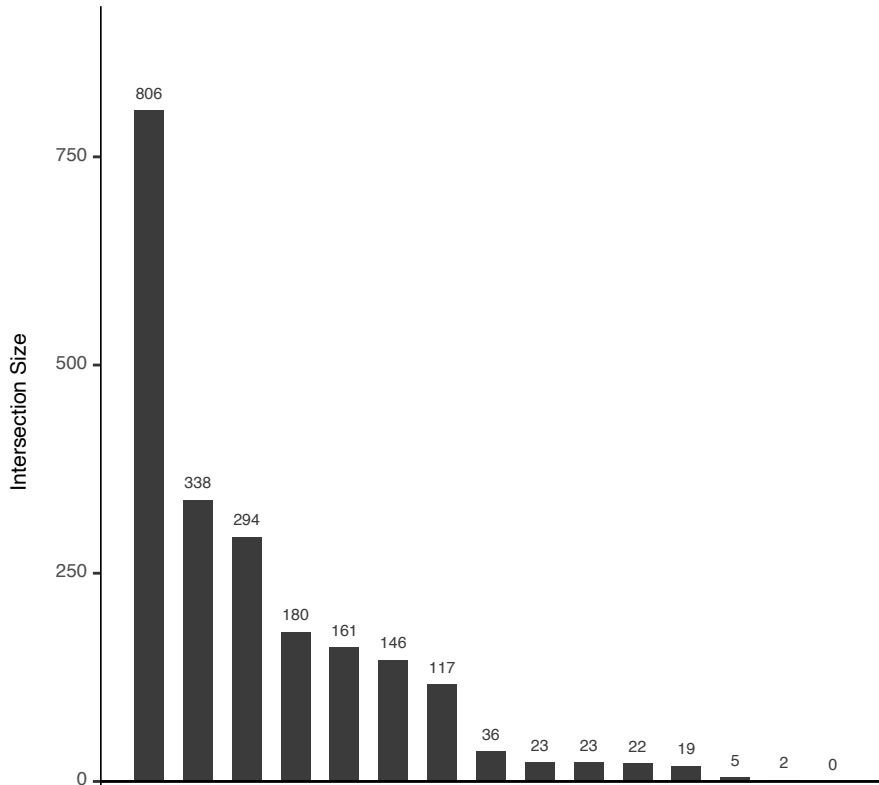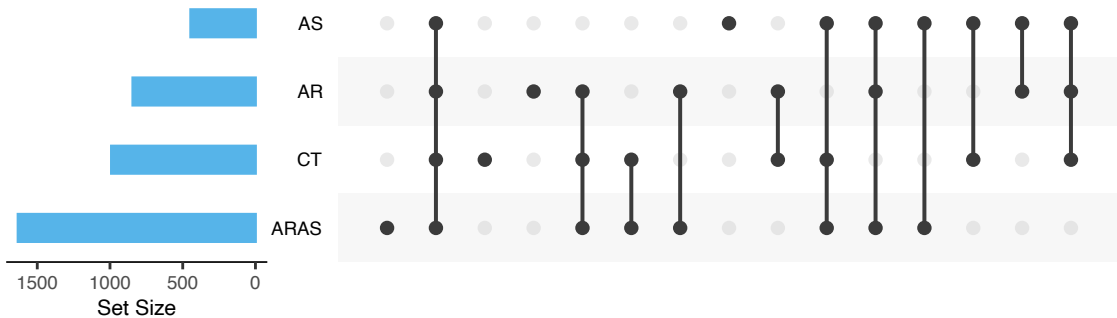

Supplement: SUPPLEMENTARY FIGURE 2 — UpSet plots of amplicon sequence variants (ASVs) in the oral bacteriomes of participants with allergic rhinitis (AR), AR with comorbid asthma (ARAS), asthma (AS) and healthy controls (CT). [file Data_Sheet_2.PDF]

## AR vs CT

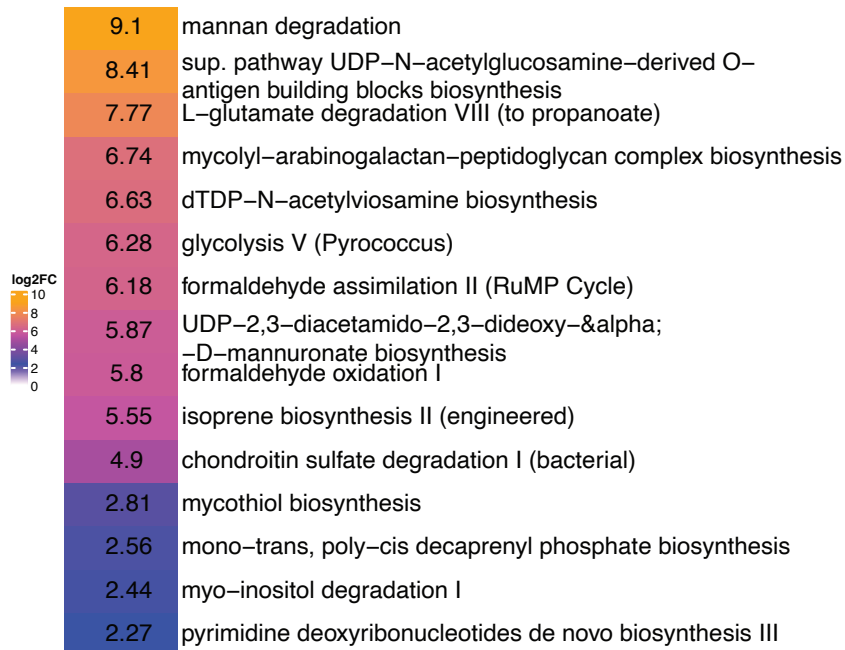

## ARAS vs CT

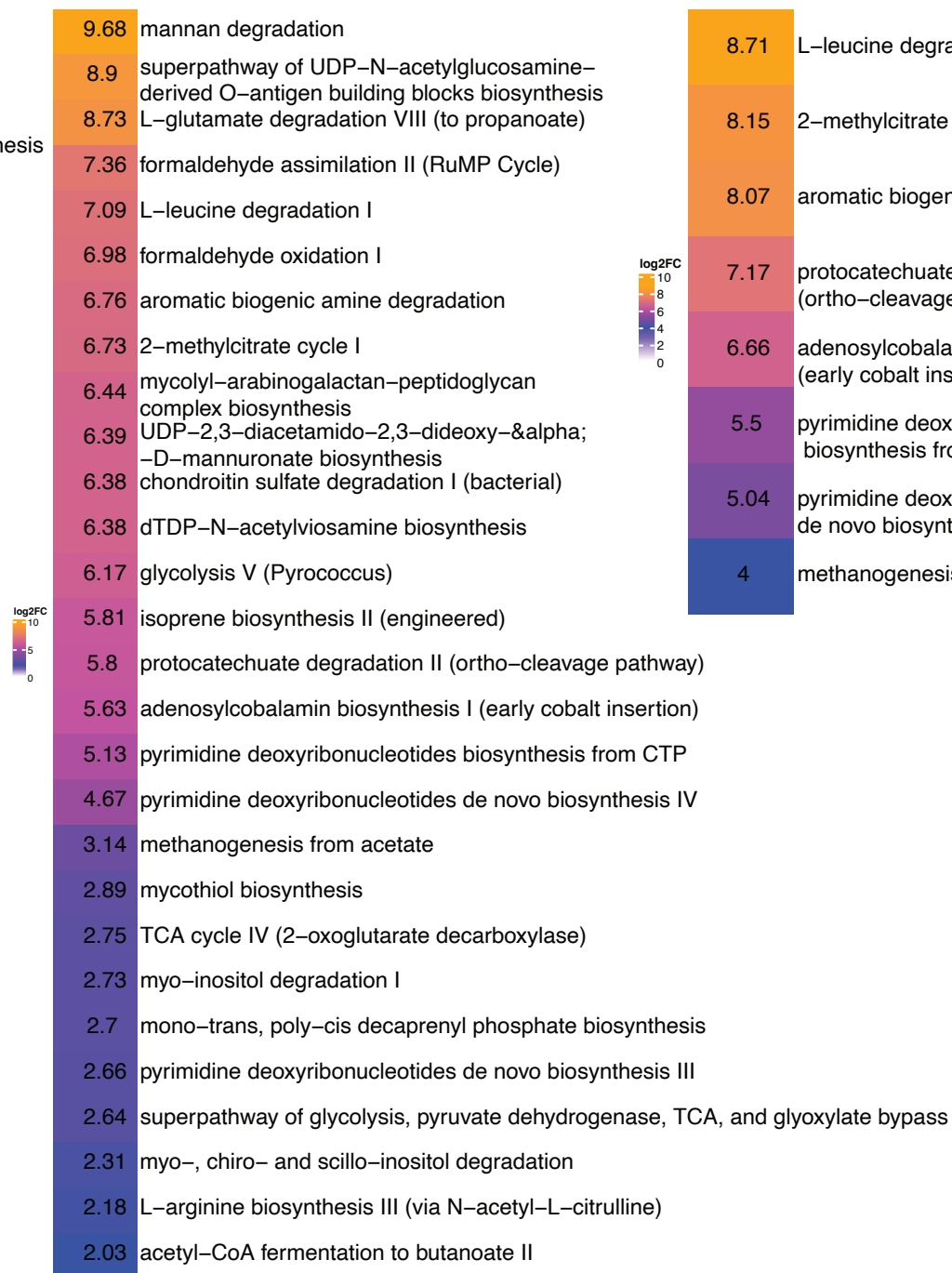

## ARAS vs AR

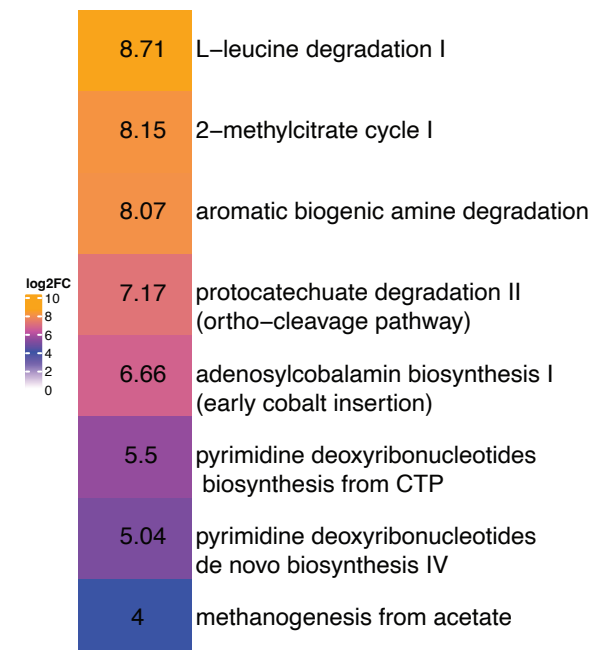

Supplement: SUPPLEMENTARY FIGURE 3 — Differential abundance analysis (Wald’s test) of functional profiles in the oral bacteriomes of participants with allergic rhinitis (AR), AR with comorbid asthma (ARAS) and healthy controls (CT). [file Data_Sheet_3.PDF]
