# Supplementary material for: The oral bacteriomes of patients with allergic rhinitis and asthma differ from that of healthy controls
Source: Front Microbiol. 2023 Jun 7;14:1197135. doi: 10.3389/fmicb.2023.1197135 (PMC10335798; doi:10.3389/fmicb.2023.1197135)
Supplement: SUPPLEMENTARY FIGURE 1 — Full account of statistical analysis performed in R. [file Data_Sheet_1.pdf]

Oral bacteriomes

2022-12-27

- Packages loading
- Filter samples and ASVs
- Core Microbiome
- Data normalization
- Estimate alpha-diversity including pd
- Estimate beta-diversity
- PCoA plots
- Statistical analyses of taxa % and other variables
- PICRUSt2 commands
  - From these last objects, prepare log2foldchange vectors
- Arrange phyloseq objects for network analysis
- Networks calculation using neighborhood selection (mb) method
- Analyze the networks
  - Label the networks nodes by the best hit and remove unwanted characters
- Plot oral bacteriome networks and color nodes by clusters/modules

Packages loading

```
library(phyloseq)
library(cowplot)
library(picante)
library(TSA)
library(multcomp)
library(microbiome)
library(mvabund)
library(geepack)
library(doBy)
library(lattice)
library(MuMIn)
library("DESeq2")
library(tidyverse)
library(stringr)
library(fantaxtic)
```

```
readRDS(file = "oral_bacteriome.RDS") -> ps1
ps1
```

```
## phyloseq-class experiment-level object
## otu_table()   OTU Table:         [ 7406 taxa and 347 samples ]
## sample_data() Sample Data:       [ 347 samples by 40 sample variables ]
## tax_table()   Taxonomy Table:    [ 7406 taxa by 7 taxonomic ranks ]
## phy_tree()    Phylogenetic Tree: [ 7406 tips and 7405 internal nodes ]
```

Filter samples and ASVs

Remove singletons

```
ps1 <- prune_taxa(taxa_sums(ps1) > 1, ps1)
ps1
```

```
## phyloseq-class experiment-level object
## otu_table()   OTU Table:         [ 2172 taxa and 347 samples ]
## sample_data() Sample Data:       [ 347 samples by 40 sample variables ]
## tax_table()   Taxonomy Table:    [ 2172 taxa by 7 taxonomic ranks ]
## phy_tree()    Phylogenetic Tree: [ 2172 tips and 2171 internal nodes ]
```

Remove samples with less than 1000 reads

```
ps1 = prune_samples(sample_sums(ps1) >= 1000, ps1)
ps1
```

```
## phyloseq-class experiment-level object
## otu_table()   OTU Table:         [ 2172 taxa and 344 samples ]
## sample_data() Sample Data:       [ 344 samples by 40 sample variables ]
## tax_table()   Taxonomy Table:    [ 2172 taxa by 7 taxonomic ranks ]
## phy_tree()    Phylogenetic Tree: [ 2172 tips and 2171 internal nodes ]
```

```
min(colSums(otu_table(ps1)))
```

```
## [1] 7114
```

```
summarize_phyloseq(ps1)
```

```
## Compositional = NO2
```

```
## 1] Min. number of reads = 71142] Max. number of reads = 682883] Total number of reads = 74556834] Average numb
er of reads = 21673.49709302335] Median number of reads = 19726.57] Sparsity = 0.9521874598483876] Any OTU sum to
1 or less? NO8] Number of singletons = 09] Percent of OTUs that are singletons
##         (i.e. exactly one read detected across all samples)010] Number of sample variables are: 40sampleIDSRAc
odepatientpatient_adjreplicalabcountrybodydisease1disease2mock_barplotpatientNdeseqmonth_adjseason_adjyearmonthse
asonN_process_CHSJageage_cage_adjsexheightweightBMIBMI_percentilobesity_CDCasthmarhinitisasthma_rhinitis2asthma_r
hinitisasthma_rhinitis1asthma_rhinitis3asthma_or_rhinitisDermatite_atopicaTCUT_IgE_positiva_aeroalergensasthma_tr
eatmentcorticoidsalergic_asthma2
```

```
## [[1]]
## [1] "1] Min. number of reads = 7114"
##
## [[2]]
## [1] "2] Max. number of reads = 68288"
##
## [[3]]
## [1] "3] Total number of reads = 7455683"
##
## [[4]]
## [1] "4] Average number of reads = 21673.4970930233"
##
## [[5]]
## [1] "5] Median number of reads = 19726.5"
##
## [[6]]
## [1] "7] Sparsity = 0.952187459848387"
##
## [[7]]
## [1] "6] Any OTU sum to 1 or less? NO"
##
## [[8]]
## [1] "8] Number of singletons = 0"
##
## [[9]]
## [1] "9] Percent of OTUs that are singletons \n        (i.e. exactly one read detected across all samples)0"
##
## [[10]]
## [1] "10] Number of sample variables are: 40"
##
## [[11]]
##  [1] "sampleID"                       "SRAcode"                        "patient"
##  [4] "patient_adj"                    "replica"                        "lab"
##  [7] "country"                        "body"                           "disease1"
## [10] "disease2"                       "mock_barplot"                   "patientN"
## [13] "deseq"                          "month_adj"                      "season_adj"
## [16] "year"                           "month"                          "season"
## [19] "N_process_CHSJ"                 "age"                            "age_c"
## [22] "age_adj"                        "sex"                            "height"
## [25] "weight"                         "BMI"                            "BMI_percentil"
## [28] "obesity_CDC"                    "asthma"                         "rhinitis"
## [31] "asthma_rhinitis2"               "asthma_rhinitis"                "asthma_rhinitis1"
## [34] "asthma_rhinitis3"               "asthma_or_rhinitis"             "Dermatite_atopica"
## [37] "TCUT_IgE_positiva_aeroalergens" "asthma_treatment"               "corticoids"
## [40] "alergic_asthma"
```

Core Microbiome

```
ps1.core <- core(ps1, detection = 0, prevalence = 0.9)
core.taxa <- taxa(ps1.core);core.taxa
```

```
##  [1] "ASV24" "ASV5"  "ASV9"  "ASV33" "ASV14" "ASV11" "ASV25" "ASV1"  "ASV35" "ASV8"
```

```
class(core.taxa)
```

```
## [1] "character"
```

Get the taxonomy data

```
tax.mat <- tax_table(ps1.core)
tax.df <- as.data.frame(tax.mat)
```

Add the ASVs to last column

```
tax.df$ASV <- rownames(tax.df)
```

Select taxonomy of only. Those ASVs that are core members based on the thresholds that were used.

```
core.taxa.class <- dplyr::filter(tax.df, rownames(tax.df) %in% core.taxa)
knitr::kable(head(core.taxa.class))
```

	Kingdom	Phylum	Class	Order	Family	Genus	Species	ASV
ASV24	Bacteria	Actinobacteriota	Actinobacteria	Actinomycetales	Actinomycetaceae	Actinomyces	naeslundii	ASV24
ASV5	Bacteria	Proteobacteria	Gammaproteobacteria	Enterobacterales	Pasteurellaceae	Haemophilus	parainfluenzae	ASV5
ASV9	Bacteria	Proteobacteria	Gammaproteobacteria	Enterobacterales	Pasteurellaceae	Haemophilus	haemolyticus	ASV9
ASV33	Bacteria	Proteobacteria	Gammaproteobacteria	Burkholderiales	Burkholderiaceae	Lautropia	unclassified	ASV33
ASV14	Bacteria	Firmicutes	Negativicutes	Veillonellales-Selenomonadales	Veillonellaceae	Veillonella	parvula	ASV14
ASV11	Bacteria	Firmicutes	Negativicutes	Veillonellales-Selenomonadales	Veillonellaceae	Veillonella	unclassified	ASV11

```
knitr::kable(core.taxa.class)
```

	Kingdom	Phylum	Class	Order	Family	Genus	Species	ASV
ASV24	Bacteria	Actinobacteriota	Actinobacteria	Actinomycetales	Actinomycetaceae	Actinomyces	naeslundii	ASV24
ASV5	Bacteria	Proteobacteria	Gammaproteobacteria	Enterobacterales	Pasteurellaceae	Haemophilus	parainfluenzae	ASV5
ASV9	Bacteria	Proteobacteria	Gammaproteobacteria	Enterobacterales	Pasteurellaceae	Haemophilus	haemolyticus	ASV9
ASV33	Bacteria	Proteobacteria	Gammaproteobacteria	Burkholderiales	Burkholderiaceae	Lautropia	unclassified	ASV33
ASV14	Bacteria	Firmicutes	Negativicutes	Veillonellales-Selenomonadales	Veillonellaceae	Veillonella	parvula	ASV14
ASV11	Bacteria	Firmicutes	Negativicutes	Veillonellales-Selenomonadales	Veillonellaceae	Veillonella	unclassified	ASV11
ASV25	Bacteria	Firmicutes	Bacilli	Lactobacillales	Streptococcaceae	Streptococcus	sanguinis	ASV25
ASV1	Bacteria	Firmicutes	Bacilli	Lactobacillales	Streptococcaceae	Streptococcus	oralis	ASV1
ASV35	Bacteria	Firmicutes	Bacilli	Lactobacillales	Carnobacteriaceae	Granulicatella	unclassified	ASV35
ASV8	Bacteria	Firmicutes	Bacilli	Staphylococcales	Gemellaceae	Gemella	unclassified	ASV8

Estimate read proportions for the core

```
rank_names(ps1.core, errorIfNULL=TRUE)
```

```
## [1] "Kingdom" "Phylum"  "Class"   "Order"   "Family"  "Genus"   "Species"
```

```
taxon <- tax_glom(ps1.core, taxrank = "Species")
table_all<-cbind(tax_table(taxon),otu_table(taxon))
table_all_t<-t(data.matrix(table_all))
write.csv(table_all_t,file="core_N.csv")
summarize_phyloseq(ps1)
```

```
## Compositional = NO2
```

```
## 1] Min. number of reads = 71142] Max. number of reads = 682883] Total number of reads = 74556834] Average numb
er of reads = 21673.49709302335] Median number of reads = 19726.57] Sparsity = 0.9521874598483876] Any OTU sum to
1 or less? NO8] Number of singletons = 09] Percent of OTUs that are singletons
##         (i.e. exactly one read detected across all samples)010] Number of sample variables are: 40sampleIDSRAc
odepatientpatient_adjreplicalabcountrybodydisease1disease2mock_barplotpatientNdeseqmonth_adjseason_adjyearmonthse
asonN_process_CHSJageage_cage_adjsexheightweightBMIBMI_percentilobesity_CDCasthmarhinitisasthma_rhinitis2asthma_r
hinitisasthma_rhinitis1asthma_rhinitis3asthma_or_rhinitisDermatite_atopicaTCUT_IgE_positiva_aeroalergensasthma_tr
eatmentcorticoidsalergic_asthma2
```

```
## [[1]]
## [1] "1] Min. number of reads = 7114"
##
## [[2]]
## [1] "2] Max. number of reads = 68288"
##
## [[3]]
## [1] "3] Total number of reads = 7455683"
##
## [[4]]
## [1] "4] Average number of reads = 21673.4970930233"
##
## [[5]]
## [1] "5] Median number of reads = 19726.5"
##
## [[6]]
## [1] "7] Sparsity = 0.952187459848387"
##
## [[7]]
## [1] "6] Any OTU sum to 1 or less? NO"
##
## [[8]]
## [1] "8] Number of singletons = 0"
##
## [[9]]
## [1] "9] Percent of OTUs that are singletons \n        (i.e. exactly one read detected across all samples)0"
##
## [[10]]
## [1] "10] Number of sample variables are: 40"
##
## [[11]]
##  [1] "sampleID"                       "SRAcode"                        "patient"
##  [4] "patient_adj"                    "replica"                        "lab"
##  [7] "country"                        "body"                           "disease1"
## [10] "disease2"                       "mock_barplot"                   "patientN"
## [13] "deseq"                          "month_adj"                      "season_adj"
## [16] "year"                           "month"                          "season"
## [19] "N_process_CHSJ"                 "age"                            "age_c"
## [22] "age_adj"                        "sex"                            "height"
## [25] "weight"                         "BMI"                            "BMI_percentil"
## [28] "obesity_CDC"                    "asthma"                         "rhinitis"
## [31] "asthma_rhinitis2"               "asthma_rhinitis"                "asthma_rhinitis1"
## [34] "asthma_rhinitis3"               "asthma_or_rhinitis"             "Dermatite_atopica"
## [37] "TCUT_IgE_positiva_aeroalergens" "asthma_treatment"               "corticoids"
## [40] "alergic_asthma"
```

Data normalization

```
diagdds = phyloseq_to_deseq2(ps1, ~season) # Any variable of the metadata would work to create the DESeq object
```

```
## converting counts to integer mode
```

```
## Warning in DESeqDataSet(se, design = design, ignoreRank): some variables in design formula are characters, con
verting to factors
```

Calculate geometric means

```
gm_mean = function(x, na.rm=TRUE){
  exp(sum(log(x[x > 0]), na.rm=na.rm) / length(x))
}
geoMeans = apply(counts(diagdds), 1, gm_mean)
```

Estimate size factors

```
diagdds = estimateSizeFactors(diagdds, geoMeans = geoMeans)
```

Get Normalized read counts

```
normcounts <- counts(diagdds, normalized = TRUE)
```

Round read counts

```
round(normcounts, digits = 0) -> normcountsrd
```

Transform matrix of normalized counts to phyloseq object

```
otu_table(normcountsrd, taxa_are_rows = TRUE) -> ncr
# Replace otu_table in original phyloseq object
otu_table(ps1) <- ncr
```

Estimate alpha-diversity including pd

```
otuD<-as.data.frame(t(otu_table(ps1)))
phylodiversityRAREF_Q<-pd(otuD, phy_tree(ps1), include.root=TRUE) ### Phylogenetic diversity. Include root=True t
ree rooted via midpoint
diversityRAREF_Q<-estimate_richness(ps1)
diversityRAREF_Q1<-cbind(sample_data(ps1),diversityRAREF_Q,phylodiversityRAREF_Q)
```

```
library(ggpubr)
```

```
my_comparisons <- list(c("AS", "CT"),c("AR", "CT"),c("ARAS", "CT"),c("AS", "AR"),c("AS", "ARAS"),c("AR", "ARAS"))
# List here the group pairs to compare statistically
```

```
compare_means(formula = Chao1~asthma_rhinitis1,data = diversityRAREF_Q1, method = "wilcox.test", exact= FALSE)
```

```
## # A tibble: 6 × 8
##   .y.   group1 group2           p     p.adj p.format p.signif method
##   <chr> <chr>  <chr>        <dbl>     <dbl> <chr>    <chr>    <chr>
## 1 Chao1 ARAS   CT     0.000000475 0.0000029 4.8e-07  ****     Wilcoxon
## 2 Chao1 ARAS   AR     0.0217      0.11      0.022    *        Wilcoxon
## 3 Chao1 ARAS   AS     0.692       1         0.692    ns       Wilcoxon
## 4 Chao1 CT     AR     0.0781      0.31      0.078    ns       Wilcoxon
## 5 Chao1 CT     AS     0.0857      0.31      0.086    ns       Wilcoxon
## 6 Chao1 AR     AS     0.537       1         0.537    ns       Wilcoxon
```

```
chao <- ggplot(diversityRAREF_Q1, aes(factor(asthma_rhinitis1), Chao1))
chao2<-chao + geom_boxplot(aes(fill = factor(asthma_rhinitis1)),outlier.colour = "black", outlier.size = 0.2)+ ge
om_jitter(size=0.2,shape=1)+panel_border(colour = "black", size = 0.5)+ ggtitle("Chao1 richness")+labs(y = "Chao1
richness") + stat_compare_means(mapping = NULL, comparisons = my_comparisons, hide.ns = FALSE, label = "p.signif"
,  label.x = NULL, label.y = NULL, exact =FALSE)
```

```
## [1] FALSE
```

```
compare_means(formula = Shannon~asthma_rhinitis1,data = diversityRAREF_Q1, method = "wilcox.test", exact= FALSE)
```

```
## # A tibble: 6 × 8
##   .y.     group1 group2        p  p.adj p.format p.signif method
##   <chr>   <chr>  <chr>     <dbl>  <dbl> <chr>    <chr>    <chr>
## 1 Shannon ARAS   CT     0.000438 0.0026 0.00044  ***      Wilcoxon
## 2 Shannon ARAS   AR     0.358    1      0.35782  ns       Wilcoxon
## 3 Shannon ARAS   AS     0.465    1      0.46547  ns       Wilcoxon
## 4 Shannon CT     AR     0.179    0.89   0.17874  ns       Wilcoxon
## 5 Shannon CT     AS     0.611    1      0.61128  ns       Wilcoxon
## 6 Shannon AR     AS     0.966    1      0.96628  ns       Wilcoxon
```

```
shan <- ggplot(diversityRAREF_Q1, aes(factor(asthma_rhinitis1), Shannon))
shan2<-shan + geom_boxplot(aes(fill = factor(asthma_rhinitis1)),outlier.colour = "black", outlier.size = 0.2)+ ge
om_jitter(size=0.2,shape=1)+panel_border(colour = "black", size = 0.5)+ ggtitle("Shannon diversity")+labs(y = "Sh
annon diversity") + stat_compare_means(mapping = NULL, comparisons = my_comparisons, hide.ns = FALSE, label = "p.
signif",  label.x = NULL, label.y = NULL, exact =FALSE)
```

```
## [1] FALSE
```

```
compare_means(formula = ACE~asthma_rhinitis1,data = diversityRAREF_Q1, method = "wilcox.test", exact= FALSE)
```

```
## # A tibble: 6 × 8
##   .y.   group1 group2          p     p.adj p.format p.signif method
##   <chr> <chr>  <chr>       <dbl>     <dbl> <chr>    <chr>    <chr>
## 1 ACE   ARAS   CT     0.00000106 0.0000064 1.1e-06  ****     Wilcoxon
## 2 ACE   ARAS   AR     0.0184     0.092     0.018    *        Wilcoxon
## 3 ACE   ARAS   AS     0.669      1         0.669    ns       Wilcoxon
## 4 ACE   CT     AR     0.120      0.44      0.120    ns       Wilcoxon
## 5 ACE   CT     AS     0.110      0.44      0.110    ns       Wilcoxon
## 6 ACE   AR     AS     0.520      1         0.520    ns       Wilcoxon
```

```
ACE <- ggplot(diversityRAREF_Q1, aes(factor(asthma_rhinitis1), ACE))
ACE2<- ACE + geom_boxplot(aes(fill = factor(asthma_rhinitis1)),outlier.colour = "black", outlier.size = 0.2)+ geo
m_jitter(size=0.2,shape=1)+panel_border(colour = "black", size = 0.5)+ ggtitle("ACE diversity")+labs(y = "ACE div
ersity") + stat_compare_means(mapping = NULL, comparisons = my_comparisons, hide.ns = FALSE, label = "p.signif",
label.x = NULL, label.y = NULL, exact =FALSE)
```

```
## [1] FALSE
```

```
compare_means(formula = PD~asthma_rhinitis1,data = diversityRAREF_Q1, method = "wilcox.test", exact= FALSE)
```

```
## # A tibble: 6 × 8
##   .y.   group1 group2       p  p.adj p.format p.signif method
##   <chr> <chr>  <chr>    <dbl>  <dbl> <chr>    <chr>    <chr>
## 1 PD    ARAS   CT     0.00124 0.0075 0.0012   **       Wilcoxon
## 2 PD    ARAS   AR     0.00549 0.027  0.0055   **       Wilcoxon
## 3 PD    ARAS   AS     0.892   1      0.8923   ns       Wilcoxon
## 4 PD    CT     AR     0.783   1      0.7831   ns       Wilcoxon
## 5 PD    CT     AS     0.272   0.99   0.2719   ns       Wilcoxon
## 6 PD    AR     AS     0.247   0.99   0.2468   ns       Wilcoxon
```

```
phyl <- ggplot(diversityRAREF_Q1, aes(factor(asthma_rhinitis1), PD))
phyl2<- phyl + geom_boxplot(aes(fill = factor(asthma_rhinitis1)),outlier.colour = "black", outlier.size = 0.2)+ g
eom_jitter(size=0.2,shape=1)+panel_border(colour = "black", size = 0.5)+ ggtitle("Phylogenetic diversity")+labs(y
= "Phylogenetic  diversity") + stat_compare_means(mapping = NULL, comparisons = my_comparisons, hide.ns = FALSE,
label = "p.signif",  label.x = NULL, label.y = NULL, exact =FALSE)
```

```
## [1] FALSE
```

```
plot_grid(chao2, shan2, ACE2, phyl2, nrows=2, cols=2, align = "v")
```

```
## Warning in plot_grid(chao2, shan2, ACE2, phyl2, nrows = 2, cols = 2, align = "v"): Argument 'cols' is deprecat
ed. Use 'ncol'
## instead.
```

```
## Warning: Removed 3 rows containing non-finite values (`stat_boxplot()`).
```

```
## Warning: Removed 3 rows containing non-finite values (`stat_signif()`).
```

```
## Warning: Removed 3 rows containing missing values (`geom_point()`).
```

```
## Warning in as_grob.default(plot): Cannot convert object of class numeric into a grob.
```

```
## Warning: Graphs cannot be vertically aligned unless the axis parameter is set. Placing graphs unaligned.
```

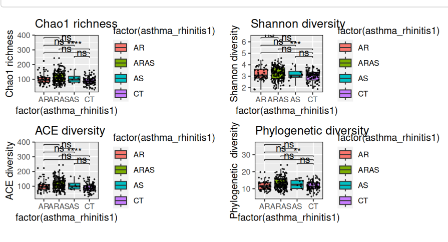

Estimate beta-diversity

Sample pairs

```
ps2 <- subset_samples(ps1, asthma_rhinitis1 == "AR" | asthma_rhinitis1 == "CT"); ps2
```

```
## phyloseq-class experiment-level object
## otu_table()   OTU Table:         [ 2172 taxa and 150 samples ]
## sample_data() Sample Data:       [ 150 samples by 40 sample variables ]
## tax_table()   Taxonomy Table:    [ 2172 taxa by 7 taxonomic ranks ]
## phy_tree()    Phylogenetic Tree: [ 2172 tips and 2171 internal nodes ]
```

```
otuD<-as.data.frame(t(otu_table(ps2)))
diversityRAREF_Q1<-cbind(sample_data(ps2))

uniun<-phyloseq::distance(ps2, method="unifrac")
uniweigh<-phyloseq::distance(ps2, method="wunifrac")
brayd<-phyloseq::distance(ps2, method="bray")
jaccd<-phyloseq::distance(ps2, method="jaccard")

t1<-adonis2(uniun~diversityRAREF_Q1$asthma_rhinitis1, perm=10000); t1
```

```
## Permutation test for adonis under reduced model
## Terms added sequentially (first to last)
## Permutation: free
## Number of permutations: 10000
##
## adonis2(formula = uniun ~ diversityRAREF_Q1$asthma_rhinitis1, permutations = 10000)
##                                     Df SumOfSqs      R2      F    Pr(>F)
## diversityRAREF_Q1$asthma_rhinitis1   1   0.8129 0.06062 9.5511 9.999e-05 ***
## Residual                           148  12.5961 0.93938
## Total                              149  13.4090 1.00000
## ---
## Signif. codes:  0 '***' 0.001 '**' 0.01 '*' 0.05 '.' 0.1 ' ' 1
```

```
t1<-adonis2(uniweigh~diversityRAREF_Q1$asthma_rhinitis1, perm=10000); t1
```

```
## Permutation test for adonis under reduced model
## Terms added sequentially (first to last)
## Permutation: free
## Number of permutations: 10000
##
## adonis2(formula = uniweigh ~ diversityRAREF_Q1$asthma_rhinitis1, permutations = 10000)
##                                     Df SumOfSqs      R2     F    Pr(>F)
## diversityRAREF_Q1$asthma_rhinitis1   1   0.4302 0.06848 10.88 9.999e-05 ***
## Residual                           148   5.8523 0.93152
## Total                              149   6.2826 1.00000
## ---
## Signif. codes:  0 '***' 0.001 '**' 0.01 '*' 0.05 '.' 0.1 ' ' 1
```

```
t1<-adonis2(brayd~diversityRAREF_Q1$asthma_rhinitis1, perm=10000); t1
```

```
## Permutation test for adonis under reduced model
## Terms added sequentially (first to last)
## Permutation: free
## Number of permutations: 10000
##
## adonis2(formula = brayd ~ diversityRAREF_Q1$asthma_rhinitis1, permutations = 10000)
##                                     Df SumOfSqs      R2      F    Pr(>F)
## diversityRAREF_Q1$asthma_rhinitis1   1    1.356 0.04097 6.3219 9.999e-05 ***
## Residual                           148   31.734 0.95903
## Total                              149   33.090 1.00000
## ---
## Signif. codes:  0 '***' 0.001 '**' 0.01 '*' 0.05 '.' 0.1 ' ' 1
```

```
t1<-adonis2(jaccd~diversityRAREF_Q1$asthma_rhinitis1, perm=10000); t1
```

```
## Permutation test for adonis under reduced model
## Terms added sequentially (first to last)
## Permutation: free
## Number of permutations: 10000
##
## adonis2(formula = jaccd ~ diversityRAREF_Q1$asthma_rhinitis1, permutations = 10000)
##                                     Df SumOfSqs      R2      F    Pr(>F)
## diversityRAREF_Q1$asthma_rhinitis1   1    1.329 0.02865 4.3653 9.999e-05 ***
## Residual                           148   45.049 0.97135
## Total                              149   46.378 1.00000
## ---
## Signif. codes:  0 '***' 0.001 '**' 0.01 '*' 0.05 '.' 0.1 ' ' 1
```

All pairs

```
uniun<-phyloseq::distance(ps1, method="unifrac")
uniweigh<-phyloseq::distance(ps1, method="wunifrac")
brayd<-phyloseq::distance(ps1, method="bray")
jaccd<-phyloseq::distance(ps1, method="jaccard")
```

PCoA plots

```
p1 = phyloseq::plot_ordination(ps1, ordinate(ps1, method="PCoA", dist="unifrac", weighted=TRUE), type = "samples"
, color = "asthma_rhinitis1") # label="patient"
p1a=p1 + geom_point(size = 2) + ggtitle("PCoA Weigthed UNIFRAC")
p2 = phyloseq::plot_ordination(ps1, ordinate(ps1, method="PCoA", dist="unifrac"), type = "samples", color = "asth
ma_rhinitis1") # label="patient"
p2a=p2 + geom_point(size = 2) + ggtitle("PCoA Unweighted UNIFRAC")
p3 = phyloseq::plot_ordination(ps1, ordinate(ps1, method="PCoA", dist="brayd"), type = "samples", color = "asthma
_rhinitis1")
p3a=p3 + geom_point(size = 2) + ggtitle("PCoA Bray-Curtis")
p4 = phyloseq::plot_ordination(ps1, ordinate(ps1, method="PCoA", dist="jaccd"), type = "samples", color = "asthma
_rhinitis1")
p4a=p4 + geom_point(size = 2) + ggtitle("PCoA Jaccard")

p1a
```

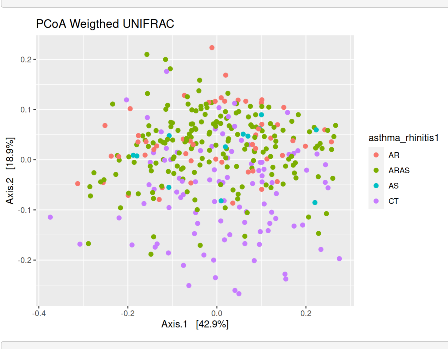


```
p2a
```

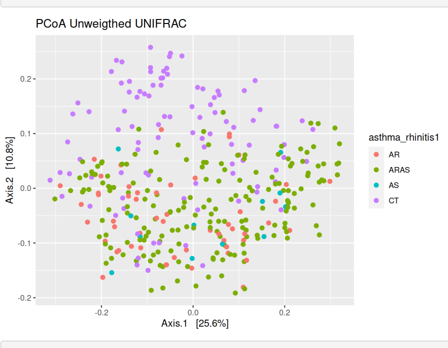


```
p3a
```

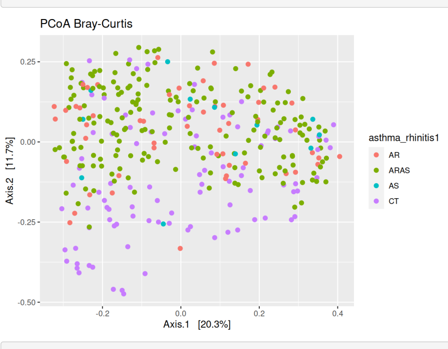


```
p4a
```

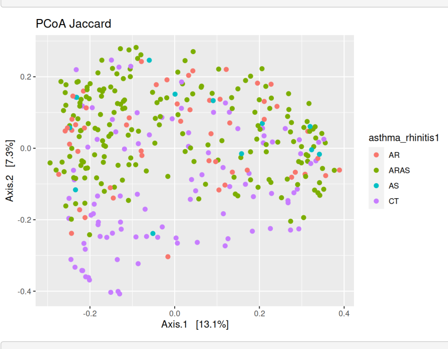


```
plot_grid(p1a, p2a, p3a, p4a, ncol = 2, nrows=2, align = "v")
```

```
## Warning in as_grob.default(plot): Cannot convert object of class numeric into a grob.
```

```
## Warning: Graphs cannot be vertically aligned unless the axis parameter is set. Placing graphs unaligned.
```

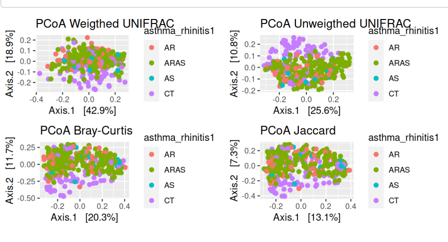

Statistical analyses of taxa % and other variables

```
bygenus <- tax_glom(ps1, taxrank = "Phylum") # ASV in taxa_lineage below = phylum name

bygenus.tr <- transform_sample_counts(bygenus, function (x) x/sum(x))
bygenus.tr.f <- filter_taxa(bygenus.tr, function (x) mean(x) > 1e-2, TRUE) # filter taxa below 5%
taxa_names(bygenus.tr.f)
```

```
## [1] "ASV30" "ASV22" "ASV24" "ASV5"  "ASV1"
```

```
taxa_lineage <- tax_table(bygenus.tr.f);taxa_lineage
```

```
## Taxonomy Table:     [5 taxa by 7 taxonomic ranks]:
##       Kingdom    Phylum             Class Order Family Genus Species
## ASV30 "Bacteria" "Fusobacteriota"   NA    NA    NA     NA    NA
## ASV22 "Bacteria" "Bacteroidota"     NA    NA    NA     NA    NA
## ASV24 "Bacteria" "Actinobacteriota" NA    NA    NA     NA    NA
## ASV5  "Bacteria" "Proteobacteria"   NA    NA    NA     NA    NA
## ASV1  "Bacteria" "Firmicutes"       NA    NA    NA     NA    NA
```

```
taxa_abun<-as.data.frame(t(otu_table(bygenus.tr.f)))
taxa_abun1<-cbind(sample_data(ps1),taxa_abun)
```

```
my_comparisons <- list(c("AS", "CT"),c("AR", "CT"),c("ARAS", "CT"),c("AS", "AR"),c("AS", "ARAS"),c("AR", "ARAS"))
# List here the group pairs to compare statistically
```

```r
library(ggpubr)
```

Run text for each dominant ASV corresponding to phyla and genera

```r
compare_means(formula = ASV30~asthma_rhinitis1, data = taxa_abun1, method = "wilcox.test", exact= FALSE)
```

```
## # A tibble: 6 × 8
##   .y.   group1 group2           p      p.adj p.format p.signif method
##   <chr> <chr>  <chr>        <dbl>      <dbl> <chr>    <chr>    <chr>
## 1 ASV30 ARAS   CT     0.0000000455 0.00000027 4.5e-08  ****     Wilcoxon
## 2 ASV30 ARAS   AR     0.832        1          0.832    ns       Wilcoxon
## 3 ASV30 ARAS   AS     0.612        1          0.612    ns       Wilcoxon
## 4 ASV30 CT     AR     0.0000777    0.00039    7.8e-05  ****     Wilcoxon
## 5 ASV30 CT     AS     0.0534       0.21       0.053    ns       Wilcoxon
## 6 ASV30 AR     AS     0.679        1          0.679    ns       Wilcoxon
```

PICRUSt2 commands

Bash section

```
place_seqs.py -s ASVs.fasta -o out.tre -p 9 --intermediate intermediate/place_seqs
```

```
hsp.py -i 16S -t ASV_tree.txt -o marker_predicted_and_nsti.tsv.gz -p 9 -n
```

```
hsp.py -i EC -t ASV_tree.txt -o marker_predicted_and_nsti.tsv.gz -p 9 -n
```

```
metagenome_pipeline.py -i ASVs.biom -m marker_predicted_and_nsti.tsv.gz -f EC_predicted.tsv.gz -o EC_metagenome_o
ut --strat_out -p 9
```

```
pathway_pipeline.py -i EC_metagenome_out/pred_metagenome_contrib.tsv.gz -o pathways_out -p 9
```

```
add_descriptions.py -i EC_metagenome_out/pred_metagenome_unstrat.tsv.gz -m EC -o EC_metagenome_out/pred_metagenom
e_unstrat_descrip.tsv.gz
```

```
add_descriptions.py -i pathways_out/path_abun_unstrat.tsv.gz -m METACYC -o pathways_out/path_abun_unstrat_descrip
.tsv.gz
```

End of bash section

```r
library("data.table")
library("ComplexHeatmap")
library("RColorBrewer")
library("circlize")
```

Laod pathway table

```r
pws_table <- fread("path_abun_unstrat_descrip.tsv.gz")
```

Load phyloseq object

```r
oral_CT_ps <- read_rds("pathway_CT.RDS")
oral_AR_ps <- read_rds("pathway_AR.RDS")
oral_ARAS_ps <- read_rds("pathway_ARAS.RDS")
```

```r
merged_oral <- merge_phyloseq(oral_CT_ps, oral_AR_ps, oral_ARAS_ps)

sample_data(merged_oral)$sample_code <- gsub("_S.+$","",sample_data(merged_oral)$sample_code)
```

```r
my_design <- ~ sex + age + asthma_rhinitis
```

```r
my_pw_counts_oral <- pws_table %>%
  dplyr::select(pathway, description, sample_data(merged_oral)$sample_code)


only_cs_my_pw_oral <- my_pw_counts_oral[,-c(1:2)]


ds_obj_oral <- DESeqDataSetFromMatrix(countData = round(only_cs_my_pw_oral),
                                      colData = sample_data(merged_oral),
                                      design = my_design)
```

```
## Warning in class(from) <- NULL: Configuración de clase (x) a NULL; resultado ya no será un objeto S4
```

```
## converting counts to integer mode
```

```
## Warning in DESeqDataSet(se, design = design, ignoreRank): some variables in design formula are characters, con
verting to factors
```

```
##   the design formula contains one or more numeric variables with integer values,
##   specifying a model with increasing fold change for higher values.
##   did you mean for this to be a factor? if so, first convert
##   this variable to a factor using the factor() function
```

```
##   the design formula contains one or more numeric variables that have mean or
##   standard deviation larger than 5 (an arbitrary threshold to trigger this message).
##   Including numeric variables with large mean can induce collinearity with the intercept.
##   Users should center and scale numeric variables in the design to improve GLM convergence.
```

```r
ds_oral_analysis <- DESeq(ds_obj_oral)
```

```
## estimating size factors
```

```
## estimating dispersions
```

```
## gene-wise dispersion estimates
```

```
## mean-dispersion relationship
```

```
## -- note: fitType='parametric', but the dispersion trend was not well captured by the
##    function: y = a/x + b, and a local regression fit was automatically substituted.
##    specify fitType='local' or 'mean' to avoid this message next time.
```

```
## final dispersion estimates
```

```
## fitting model and testing
```

```
## -- replacing outliers and refitting for 28 genes
## -- DESeq argument 'minReplicatesForReplace' = 7
## -- original counts are preserved in counts(dds)
```

```
## estimating dispersions
```

```
## fitting model and testing
```

```r
ARAS_vs_CT_wald <- results(ds_oral_analysis, contrast = c("asthma_rhinitis", "AR", "NO"))

AR_vs_CT_wald <- results(ds_oral_analysis, contrast = c("asthma_rhinitis", "RN", "NO"))

ARAS_vs_AR_wald <- results(ds_oral_analysis, contrast = c("asthma_rhinitis", "AR", "RN"))
```

Subset for p-value < 0.05

```r
oral_ARAS_CT_sigres <- ARAS_vs_CT_wald %>%
  data.frame() %>%
  rownames_to_column(var = "pathway") %>%
  as_tibble() %>%
  dplyr::filter(padj < 0.05)
```

```r
oral_AR_CT_sigres <- AR_vs_CT_wald %>%
  data.frame() %>%
  rownames_to_column(var = "pathway") %>%
  as_tibble() %>%
  dplyr::filter(padj < 0.05)
```

```r
oral_ARAS_vs_AR_sigres <- ARAS_vs_AR_wald %>%
  data.frame() %>%
  rownames_to_column(var = "pathway") %>%
  as_tibble() %>%
  dplyr::filter(padj < 0.05)
```

```r
oral_ARAS_CT_sigres$pathway <- pws_table$description[as.numeric(oral_ARAS_CT_sigres$pathway)]


oral_ARAS_CT_sigres <- oral_ARAS_CT_sigres[order(oral_ARAS_CT_sigres$log2FoldChange, decreasing = TRUE),]


oral_ARAS_CT_sigres <- oral_ARAS_CT_sigres[abs(oral_ARAS_CT_sigres$log2FoldChange) >= 2,]


oral_ARAS_CT_sigres
```

```
## # A tibble: 28 × 7
##    pathway                                                                     baseM…¹ log2F…² lfcSE  st
at    pvalue      padj
##    <chr>                                                                         <dbl>   <dbl> <dbl> <db
l>     <dbl>     <dbl>
##  1 mannan degradation                                                            184.     9.68 0.321  30
.2 4.72e-200 1.35e-197
##  2 superpathway of UDP-N-acetylglucosamine-derived O-antigen building blocks biosyn…   104.     8.90 0.337  26
.4 2.40e-153 3.43e-151
##  3 L-glutamate degradation VIII (to propanoate)                                   91.9    8.73 0.349  25
.0 2.23e-138 2.13e-136
##  4 formaldehyde assimilation II (RuMP Cycle)                                      63.3    7.36 0.511  14
.4 5.76e- 47 2.35e- 45
##  5 L-leucine degradation I                                                        29.7    7.09 0.592  12
.0 5.16e- 33 8.19e- 32
##  6 formaldehyde oxidation I                                                       46.6    6.98 0.506  13
.8 2.75e- 43 7.87e- 42
##  7 aromatic biogenic amine degradation (bacteria)                                 18.5    6.76 0.553  12
.2 2.68e- 34 4.78e- 33
##  8 2-methylcitrate cycle I                                                        19.7    6.73 0.482  14
.0 2.27e- 44 7.21e- 43
##  9 mycolyl-arabinogalactan-peptidoglycan complex biosynthesis                     36.2    6.44 0.334  19
.3 1.18e- 82 8.47e- 81
## 10 UDP-2,3-diacetamido-2,3-dideoxy-&alpha;-D-mannuronate biosynthesis             14.9    6.39 0.430  14
.8 7.27e- 50 3.47e- 48
## # … with 18 more rows, and abbreviated variable names ¹baseMean, ²log2FoldChange
```

```r
oral_AR_CT_sigres$pathway <- pws_table$description[as.numeric(oral_AR_CT_sigres$pathway)]

oral_AR_CT_sigres <- oral_AR_CT_sigres[order(oral_AR_CT_sigres$log2FoldChange, decreasing = TRUE),]

oral_AR_CT_sigres <- oral_AR_CT_sigres[abs(oral_AR_CT_sigres$log2FoldChange) >= 2,]

oral_AR_CT_sigres
```

```
## # A tibble: 15 × 7
##    pathway                                                                     baseM…¹ log2F…² lfcSE  st
at    pvalue      padj
##    <chr>                                                                         <dbl>   <dbl> <dbl> <db
l>     <dbl>     <dbl>
##  1 mannan degradation                                                            184.     9.10 0.372 24.
4  5.36e-132 1.53e-129
##  2 superpathway of UDP-N-acetylglucosamine-derived O-antigen building blocks biosyn…   104.     8.41 0.395 21.
3  7.52e-101 1.08e- 98
##  3 L-glutamate degradation VIII (to propanoate)                                   91.9    7.77 0.410 18.
9  5.83e- 80 5.56e- 78
##  4 mycolyl-arabinogalactan-peptidoglycan complex biosynthesis                     36.2    6.74 0.395 17.
1  3.10e- 65 2.22e- 63
##  5 dTDP-N-acetylviosamine biosynthesis                                            15.7    6.63 0.650 10.
2  2.17e- 24 6.89e- 23
##  6 glycolysis V (Pyrococcus)                                                      17.7    6.28 0.576 10.
9  1.07e- 27 4.38e- 26
##  7 formaldehyde assimilation II (RuMP Cycle)                                      63.3    6.18 0.630  9.
81 1.03e- 22 2.68e- 21
##  8 UDP-2,3-diacetamido-2,3-dideoxy-&alpha;-D-mannuronate biosynthesis             14.9    5.87 0.518 11.
3  1.07e- 29 5.09e- 28
##  9 formaldehyde oxidation I                                                       46.6    5.80 0.623  9.
31 1.34e- 20 2.73e- 19
## 10 isoprene biosynthesis II (engineered)                                          19.2    5.55 0.553 10.
0  1.03e- 23 2.95e- 22
## 11 chondroitin sulfate degradation I (bacterial)                                  13.7    4.90 0.423 11.
6  4.30e- 31 2.46e- 29
## 12 mycothiol biosynthesis                                                        128.     2.81 0.293  9.
59 8.79e- 22 2.09e- 20
## 13 mono-trans, poly-cis decaprenyl phosphate biosynthesis                        376.     2.56 0.242 10.
6  3.60e- 26 1.29e- 24
## 14 myo-inositol degradation I                                                     94.6    2.44 0.260  9.
36 8.06e- 21 1.77e- 19
## 15 pyrimidine deoxyribonucleotides de novo biosynthesis III                      419.     2.27 0.352  6.
46 1.05e- 10 1.42e-  9
## # … with abbreviated variable names ¹baseMean, ²log2FoldChange
```

```r
oral_ARAS_vs_AR_sigres$pathway <- pws_table$description[as.numeric(oral_ARAS_vs_AR_sigres$pathway)]
oral_ARAS_vs_AR_sigres <- oral_ARAS_vs_AR_sigres[order(oral_ARAS_vs_AR_sigres$log2FoldChange,
                                                       decreasing = T),]

oral_ARAS_vs_AR_sigres <- oral_ARAS_vs_AR_sigres[abs(oral_ARAS_vs_AR_sigres$log2FoldChange) >= 2,]
oral_ARAS_vs_AR_sigres
```

```
## # A tibble: 8 × 7
##   pathway                                               baseMean log2FoldChange lfcSE  stat   pvalue     p
adj
##   <chr>                                                    <dbl>          <dbl> <dbl> <dbl>    <dbl>    <d
bl>
## 1 L-leucine degradation I                                  29.7            8.71 0.603 14.4  3.11e-47 4.44e
-45
## 2 2-methylcitrate cycle I                                  19.7            8.15 0.498 16.4  2.90e-60 8.30e
-58
## 3 aromatic biogenic amine degradation (bacteria)           18.5            8.07 0.564 14.3  1.86e-46 1.77e
-44
## 4 protocatechuate degradation II (ortho-cleavage pathway)   9.79           7.17 0.728  9.84 7.23e-23 5.17e
-21
## 5 adenosylcobalamin biosynthesis I (early cobalt insertion)  6.99          6.66 1.02   6.53 6.43e-11 2.04e
- 9
## 6 pyrimidine deoxyribonucleotides biosynthesis from CTP     3.61           5.50 0.745  7.38 1.59e-13 9.08e
-12
## 7 pyrimidine deoxyribonucleotides de novo biosynthesis IV   2.54           5.04 0.728  6.92 4.53e-12 1.85e
-10
## 8 methanogenesis from acetate                               1.04           4.00 0.583  6.87 6.52e-12 2.33e
-10
```

From these last objects, prepare log2foldchange vectors

Log2fc for ARAS vs CT

```r
log2fc_oral_ARAS_CT <- oral_ARAS_CT_sigres$log2FoldChange %>%
  as.matrix()


colnames(log2fc_oral_ARAS_CT) <- "log2FC"
```

```r
log2fc_oral_ARAS_CT_colors <- colorRamp2(c(min(log2fc_oral_ARAS_CT),0,max(log2fc_oral_ARAS_CT)),
                                         c("blue","white","orange"))



hm_oral_CT_ARAS_fc <- Heatmap(log2fc_oral_ARAS_CT, cluster_rows = F, row_labels = oral_ARAS_CT_sigres$pathway,
                              col = log2fc_oral_ARAS_CT_colors, width = unit(30,"mm"),
                              cell_fun = function(j,i,x,y,w,h,col){
                                grid.text(round(log2fc_oral_ARAS_CT[i,j],2),x,y)
                              }, name = "log2FC", column_labels = "")



draw(hm_oral_CT_ARAS_fc, heatmap_legend_side = "left")
```

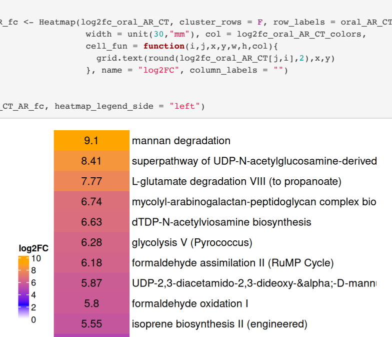

Log2fc for AR and CT

```r
log2fc_oral_AR_CT <- oral_AR_CT_sigres$log2FoldChange %>%
  as.matrix()

colnames(log2fc_oral_AR_CT) <- "log2FC"

log2fc_oral_AR_CT_colors <- colorRamp2(c(min(log2fc_oral_AR_CT),0,max(log2fc_oral_AR_CT)),
                                       c("blue","white","orange"))


hm_oral_CT_AR_fc <- Heatmap(log2fc_oral_AR_CT, cluster_rows = F, row_labels = oral_AR_CT_sigres$pathway,
                            width = unit(30,"mm"), col = log2fc_oral_AR_CT_colors,
                            cell_fun = function(i,j,x,y,w,h,col){
                              grid.text(round(log2fc_oral_AR_CT[j,i],2),x,y)
                            }, name = "log2FC", column_labels = "")


draw(hm_oral_CT_AR_fc, heatmap_legend_side = "left")
```

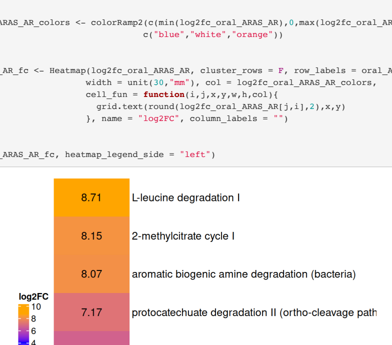

Log2fc for ARAS vs AR

```r
log2fc_oral_ARAS_AR <- oral_ARAS_vs_AR_sigres$log2FoldChange %>%
  as.matrix()

colnames(log2fc_oral_ARAS_AR) <- "log2FC"


log2fc_oral_ARAS_AR_colors <- colorRamp2(c(min(log2fc_oral_ARAS_AR),0,max(log2fc_oral_ARAS_AR)),
                                         c("blue","white","orange"))


hm_oral_ARAS_AR_fc <- Heatmap(log2fc_oral_ARAS_AR, cluster_rows = F, row_labels = oral_ARAS_vs_AR_sigres$pathway,
                              width = unit(30,"mm"), col = log2fc_oral_ARAS_AR_colors,
                              cell_fun = function(i,j,x,y,w,h,col){
                                grid.text(round(log2fc_oral_ARAS_AR[j,i],2),x,y)
                              }, name = "log2FC", column_labels = "")


draw(hm_oral_ARAS_AR_fc, heatmap_legend_side = "left")
```

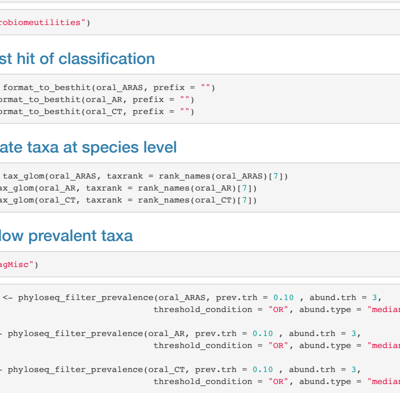

Arrange phyloseq objects for network analysis

```r
oral_ARAS <- read_rds("pathway_ARAS.RDS")
oral_AR <- read_rds("pathway_AR.RDS")
oral_CT <- read_rds("pathway_CT.RDS")
```

```r
library("microbiomeutilities")
```

Define best hit of classification

```r
oral_ARAS <- format_to_besthit(oral_ARAS, prefix = "")
oral_AR <- format_to_besthit(oral_AR, prefix = "")
oral_CT <- format_to_besthit(oral_CT, prefix = "")
```

Agglomerate taxa at species level

```r
oral_ARAS <- tax_glom(oral_ARAS, taxrank = rank_names(oral_ARAS)[7])
oral_AR <- tax_glom(oral_AR, taxrank = rank_names(oral_AR)[7])
oral_CT <- tax_glom(oral_CT, taxrank = rank_names(oral_CT)[7])
```

Filter out low prevalent taxa

```r
library("metagMisc")
```

```r
oral_ARASprv <- phyloseq_filter_prevalence(oral_ARAS, prev.trh = 0.10 , abund.trh = 3,
                                           threshold_condition = "OR", abund.type = "median")

oral_ARprv <- phyloseq_filter_prevalence(oral_AR, prev.trh = 0.10 , abund.trh = 3,
                                         threshold_condition = "OR", abund.type = "median")

oral_CTprv <- phyloseq_filter_prevalence(oral_CT, prev.trh = 0.10 , abund.trh = 3,
                                         threshold_condition = "OR", abund.type = "median" )
```

Remove non prokaryotic ASVs

```r
taxa_forfilter <- c("Chloroplast", "Mitochondria","Eukaryota")

ps_list = list(oral_ARASprv, oral_ARprv, oral_CTprv)

for (i in 1:length(ps_list)) {
  ps_list[[i]] <- subset_taxa(ps_list[[i]],
                              !Domain %in% taxa_forfilter &
                                !Phylum %in% taxa_forfilter &
                                !Class %in% taxa_forfilter &
                                !Order %in% taxa_forfilter &
                                !Family %in% taxa_forfilter &
                                !Genus %in% taxa_forfilter )   }

oralARAS <- ps_list[[1]]
oralAR <- ps_list[[2]]
oralCT <- ps_list[[3]]
```

```r
library("SpiecEasi")
```

Networks calculation using neighborhood selection (mb) method

```r
oralCT_net = spiec.easi(oralCT, method = "mb", nlambda = 20,
                        lambda.min.ratio = 1e-2, pulsar.params = list(rep.num=50,
                                                                      ncores=11))
```

```
## Applying data transformations...
```

```
## Selecting model with pulsar using stars...
```

```
## Fitting final estimate with mb...
```

```
## done
```

```r
oralARAS_net = spiec.easi(oralARAS, method = "mb", nlambda = 20,
                          lambda.min.ratio = 1e-2, pulsar.params = list(rep.num=50,
                                                                        ncores=11))
```

```
## Applying data transformations...
```

```
## Selecting model with pulsar using stars...
```

```
## Fitting final estimate with mb...
```

```
## done
```

```r
oralAR_net = spiec.easi(oralAR, method = "mb", nlambda = 20,
                        lambda.min.ratio = 1e-2, pulsar.params = list(rep.num=50,
                                                                      ncores=11))
```

```
## Applying data transformations...
```

```
## Selecting model with pulsar using stars...
```

```
## Fitting final estimate with mb...
```

```
## done
```

```r
# the resulting variables were stored in RDS format and exported for local analysis
```

Retrieve adjacency matrices

```r
library("NetCoMi")
```

```r
adj_oralARAS <- symBeta(getOptBeta(oralARAS_net), mode = "maxabs") %>%
  as.matrix()

rownames(adj_oralARAS) <- colnames(otu_table(oralARAS))
colnames(adj_oralARAS) <- colnames(otu_table(oralARAS))
```

```r
adj_oralAR <- symBeta(getOptBeta(oralAR_net), mode = "maxabs") %>%
  as.matrix()

rownames(adj_oralAR) <- colnames(otu_table(oralAR))
colnames(adj_oralAR) <- colnames(otu_table(oralAR))
```

```r
adj_oralCT <- symBeta(getOptBeta(oralCT_net), mode = "maxabs") %>%
  as.matrix()

rownames(adj_oralCT) <- colnames(otu_table(oralCT))
colnames(adj_oralCT) <- colnames(otu_table(oralCT))
```

Construct the networks

```r
oralARAS_netcomi <- netConstruct(data = adj_oralARAS,
                                 normMethod = "none", zeroMethod = "none",
                                 sparsMethod = "none", dataType = "condDependence",
                                 verbose = 3, seed = 1234)
```

```
## Checking input arguments ... Done.
```

```r
oralAR_netcomi <- netConstruct(data = adj_oralAR,
                               normMethod = "none", zeroMethod = "none",
                               sparsMethod = "none", dataType = "condDependence",
                               verbose = 3, seed = 1234)
```

```
## Checking input arguments ... Done.
```

```r
oralCT_netcomi <- netConstruct(data = adj_oralCT,
                               normMethod = "none", zeroMethod = "none",
                               sparsMethod = "none", dataType = "condDependence",
                               verbose = 3, seed = 1234)
```

```
## Checking input arguments ... Done.
```

Analyze the networks

```r
oralARAS_analyzed <- netAnalyze(oralARAS_netcomi, centrLCC = FALSE, avDissIgnoreInf = TRUE,
                                hubPar = "degree", hubQuant = 0.90,
                                normDeg = TRUE, normBetw = TRUE, normEigen = TRUE, verbose = 2)
```

```
## Checking input arguments ... Done.
## Create igraph objects ... Done.
## Compute clustering (cluster_fast_greedy) ... Done.
## Compute shortest paths ... Done.
##
## Compute global properties:
## Average dissimilarity ... Done.
## Edge / vertex connectivity ... Done.
## Natural connectivity ... Done.
## Clustering coefficient ... Done.
## Modularity ... Done.
## Density ... Done.
## P-N-Ratio ... Done.
##
## Compute centralities:
## Degree ... Done.
## Betweenness centrality ... Done.
## Closeness centrality ... Done.
## Eigenvector centrality ... Done.
##
## Compute hubs (based on empirical quantiles) ... Done.
## Compute GCM ... Done.
```

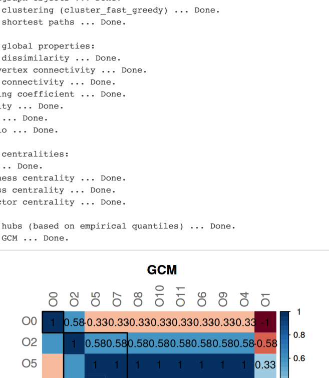


```r
oralAR_analyzed <- netAnalyze(oralAR_netcomi, centrLCC = FALSE, avDissIgnoreInf = TRUE,
                              hubPar = "degree", hubQuant = 0.90,
                              normDeg = TRUE, normBetw = TRUE, normEigen = TRUE, verbose = 2)
```

```
## Checking input arguments ... Done.
## Create igraph objects ... Done.
## Compute clustering (cluster_fast_greedy) ... Done.
## Compute shortest paths ... Done.
##
## Compute global properties:
## Average dissimilarity ... Done.
## Edge / vertex connectivity ... Done.
## Natural connectivity ... Done.
## Clustering coefficient ... Done.
## Modularity ... Done.
## Density ... Done.
## P-N-Ratio ... Done.
##
## Compute centralities:
## Degree ... Done.
## Betweenness centrality ... Done.
## Closeness centrality ... Done.
## Eigenvector centrality ... Done.
##
## Compute hubs (based on empirical quantiles) ... Done.
## Compute GCM ... Done.
```

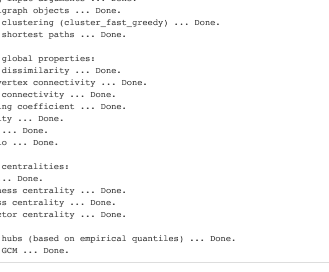


```r
oralCT_analyzed <- netAnalyze(oralCT_netcomi, centrLCC = FALSE, avDissIgnoreInf = TRUE,
                              hubPar = "degree", hubQuant = 0.90,
                              normDeg = TRUE, normBetw = TRUE, normEigen = TRUE, verbose = 2)
```

```
## Checking input arguments ... Done.
## Create igraph objects ... Done.
## Compute clustering (cluster_fast_greedy) ... Done.
## Compute shortest paths ... Done.
##
## Compute global properties:
## Average dissimilarity ... Done.
## Edge / vertex connectivity ... Done.
## Natural connectivity ... Done.
## Clustering coefficient ... Done.
## Modularity ... Done.
## Density ... Done.
## P-N-Ratio ... Done.
##
## Compute centralities:
## Degree ... Done.
## Betweenness centrality ... Done.
## Closeness centrality ... Done.
## Eigenvector centrality ... Done.
##
## Compute hubs (based on empirical quantiles) ... Done.
## Compute GCM ... Done.
```

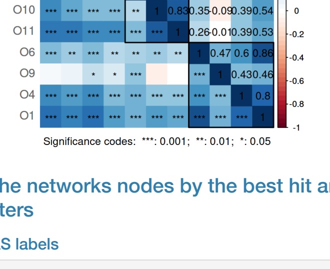

Label the networks nodes by the best hit and remove unwanted characters

oral ARAS labels

```r
tax_oralARAS <- tax_table(oralARAS) %>%
  as.data.frame() %>%
  dplyr::mutate(lab = paste(Genus, Species, sep = " ")) %>%
  dplyr::select(-best_hit)

tax_oralARAS$lab <- gsub("g__.+$", "", tax_oralARAS$lab)
tax_oralARAS$lab <- gsub("f__.*?\\s", "", tax_oralARAS$lab)
```

```
tax_oralARAS$lab <- gsub("f__","", tax_oralARAS$lab)
tax_oralARAS$lab <- gsub("o__.*?\\s", "", tax_oralARAS$lab)
tax_oralARAS$lab <- gsub("o__", "", tax_oralARAS$lab)


labels_oralARAS <- tax_oralARAS$lab
names(labels_oralARAS) <- rownames(tax_oralARAS)
```

oral AR labels

```
tax_oralAR <- tax_table(oralAR) %>%
  as.data.frame() %>%
  dplyr::mutate(lab = paste(Genus, Species, sep = " ")) %>%
  dplyr::select(-best_hit)

tax_oralAR$lab <- gsub("g__.+$", "", tax_oralAR$lab)
tax_oralAR$lab <- gsub("f__.*?\\s", "", tax_oralAR$lab)
tax_oralAR$lab <- gsub("f__","", tax_oralAR$lab)
tax_oralAR$lab <- gsub("o__.*?\\s", "", tax_oralAR$lab)
tax_oralAR$lab <- gsub("o__", "", tax_oralAR$lab)


labels_oralAR <- tax_oralAR$lab
names(labels_oralAR) <- rownames(tax_oralAR)
```

oral CT labels

```
tax_oralCT <- tax_table(oralCT) %>%
  as.data.frame() %>%
  dplyr::mutate(lab = paste(Genus, Species, sep = " ")) %>%
  dplyr::select(-best_hit)

tax_oralCT$lab <- gsub("g__.+$", "", tax_oralCT$lab)
tax_oralCT$lab <- gsub("f__.*?\\s", "", tax_oralCT$lab)
tax_oralCT$lab <- gsub("f__","", tax_oralCT$lab)
tax_oralCT$lab <- gsub("o__.*?\\s", "", tax_oralCT$lab)
tax_oralCT$lab <- gsub("o__", "", tax_oralCT$lab)


labels_oralCT <- tax_oralCT$lab
names(labels_oralCT) <- rownames(tax_oralCT)
```

Plot oral bacteriome networks and color nodes by clusters/modules

```
oralARAS_circle <- plot(oralARAS_analyzed, cexNodes = 1.3,
                        cexHubLabels = 1.9 , cexLabels = 2.7, cexTitle = 2.5, nodeColor = "cluster",
                        cexHubs = 1.6, rmSingles = "all",
                        repulsion = 2.8,
                        layout = "layout_nicely", labels = labels_oralARAS)
```

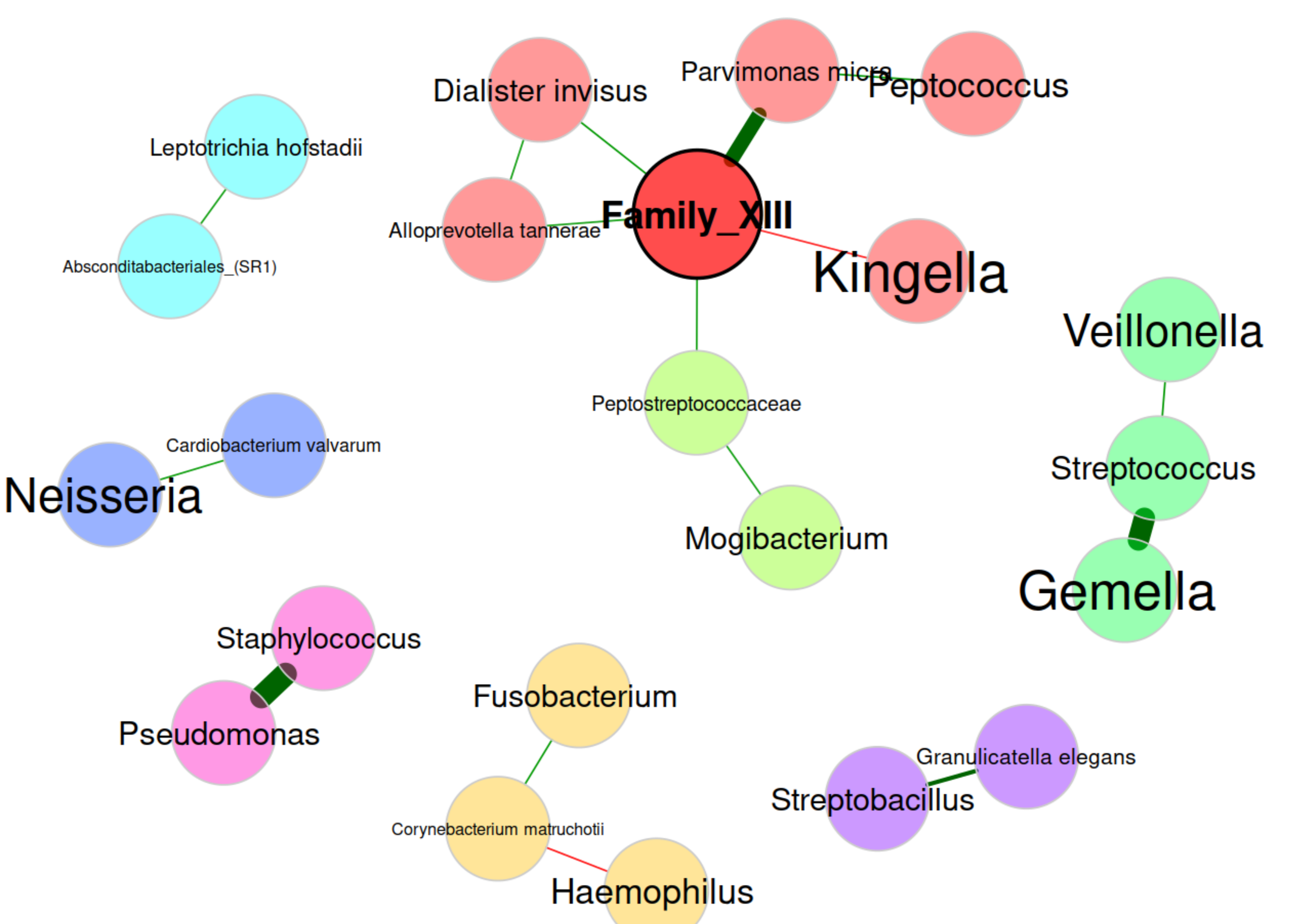


```
oralAR_circle <- plot(oralAR_analyzed, cexNodes = 1.3,
                      cexHubLabels = 1.9 , cexLabels = 2.7, cexTitle = 2.5, nodeColor = "cluster",
                      cexHubs = 1.6, rmSingles = "all",
                      repulsion = 2.8,
                      layout = "layout_nicely", labels = labels_oralAR)
```

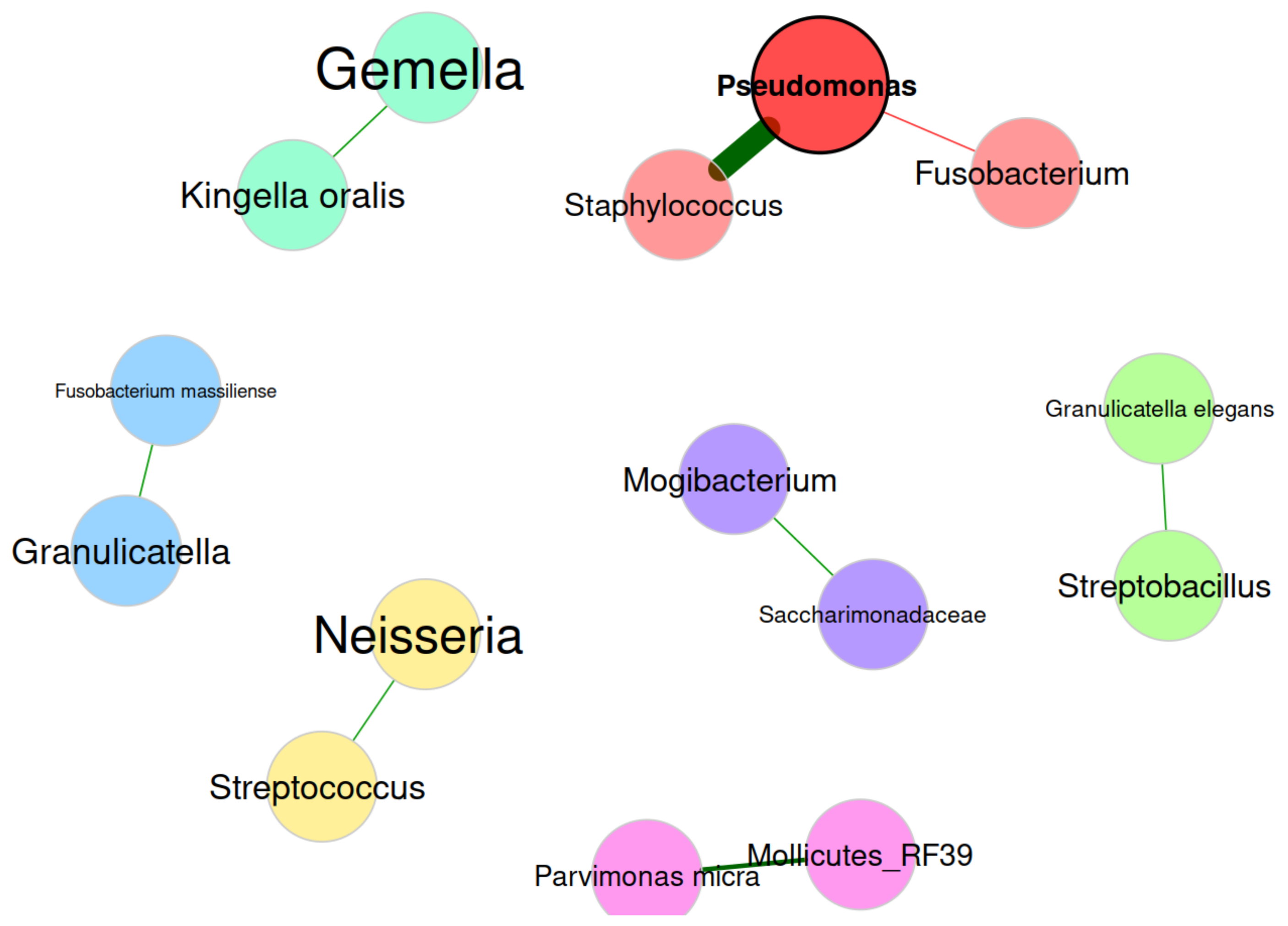


```
oralCT_circle <- plot(oralCT_analyzed, cexNodes = 1.3,
                      cexHubLabels = 1.9 , cexLabels = 2.7, cexTitle = 2.5, nodeColor = "cluster",
                      cexHubs = 1.6, rmSingles = "all",
                      repulsion = 2.8,
                      layout = "layout_nicely", labels = labels_oralCT )
```

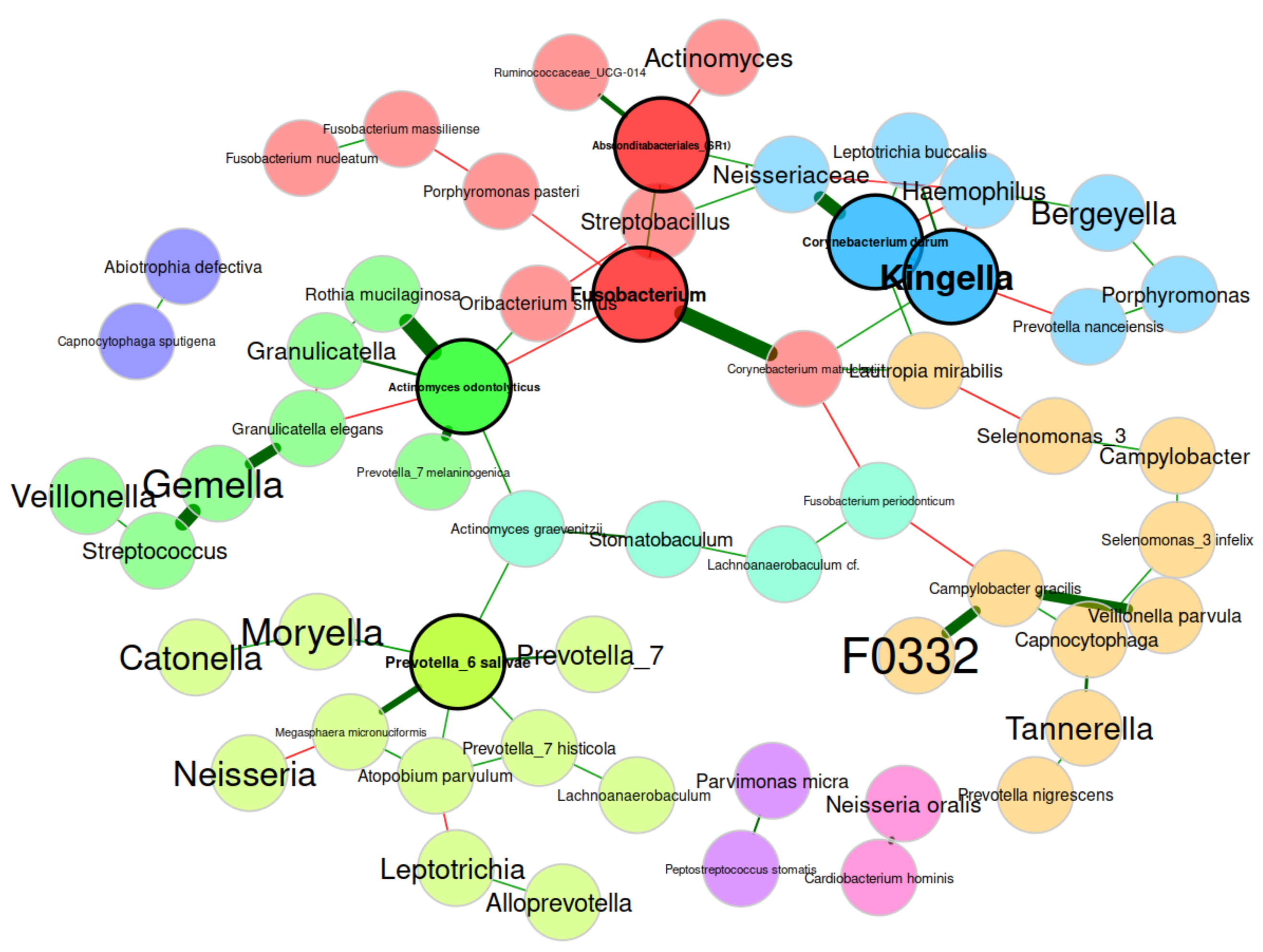

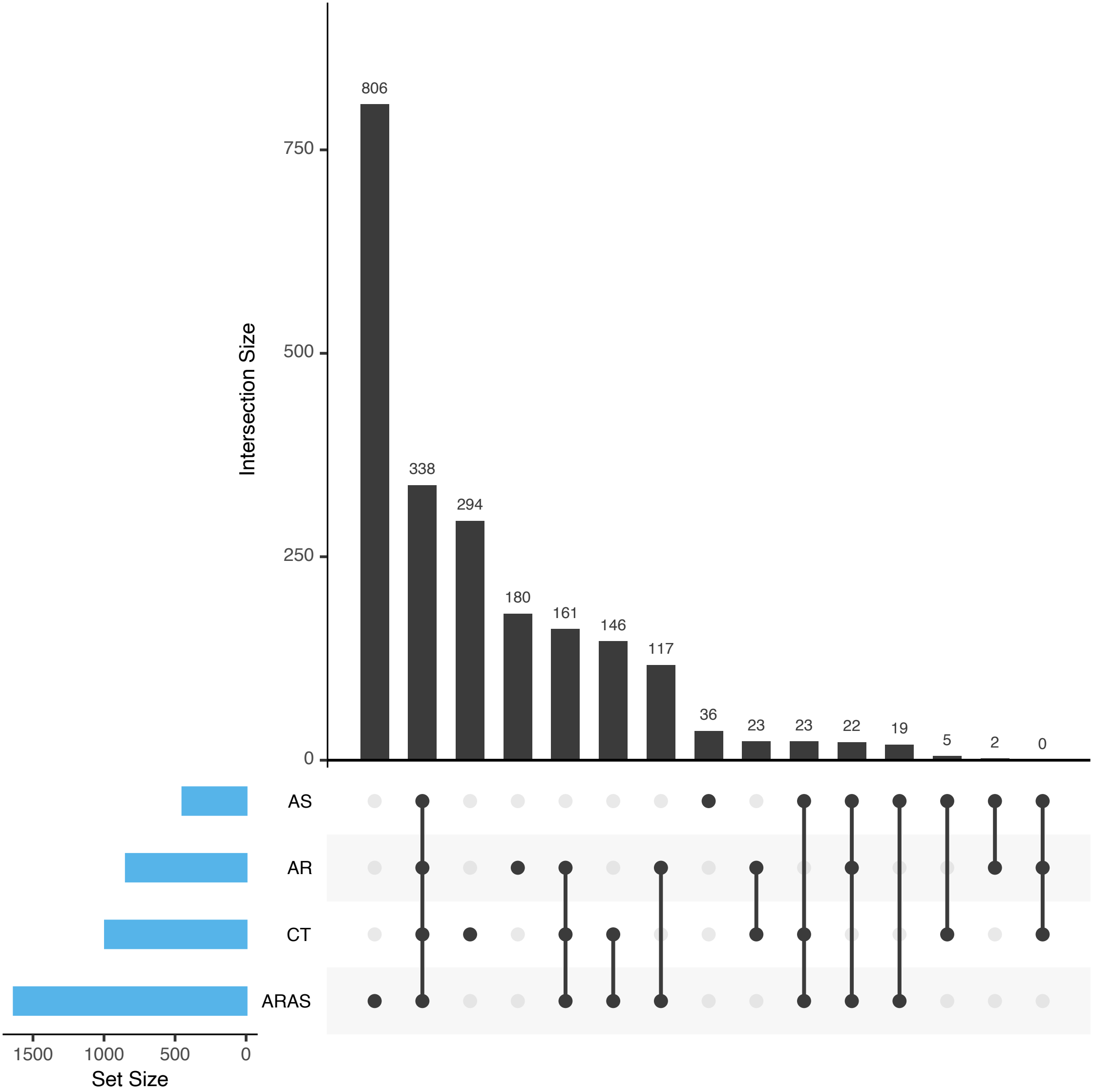

Intersection Size
750
500
250
0
806
338
294
180
161
146
117
36
23
23
22
19
5
2
0
AS
AR
CT
ARAS
1500
1000
500
0
Set Size

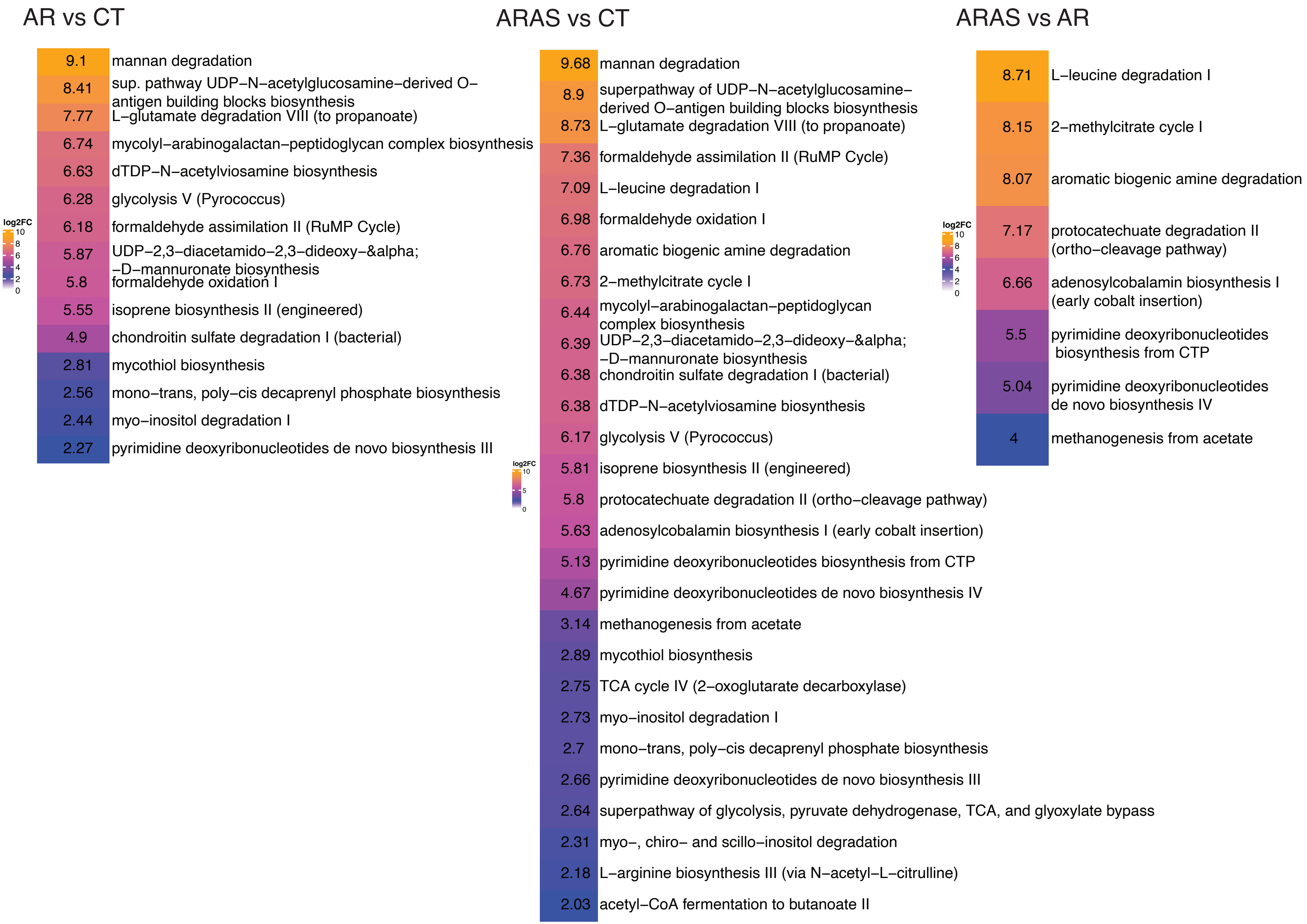
AR vs CT
9.1 mannan degradation
8.41 sup. pathway UDP−N−acetylglucosamine−derived O−antigen building blocks biosynthesis
7.77 L−glutamate degradation VIII (to propanoate)
6.74 mycolyl−arabinogalactan−peptidoglycan complex biosynthesis
6.63 dTDP−N−acetylviosamine biosynthesis
6.28 glycolysis V (Pyrococcus)
6.18 formaldehyde assimilation II (RuMP Cycle)
5.87 UDP−2,3−diacetamido−2,3−dideoxy−α−D−mannuronate biosynthesis
5.8 formaldehyde oxidation I
5.55 isoprene biosynthesis II (engineered)
4.9 chondroitin sulfate degradation I (bacterial)
2.81 mycothiol biosynthesis
2.56 mono−trans, poly−cis decaprenyl phosphate biosynthesis
2.44 myo−inositol degradation I
2.27 pyrimidine deoxyribonucleotides de novo biosynthesis III
log2FC
10
8
6
4
2
0
ARAS vs CT
9.68 mannan degradation
8.9 superpathway of UDP−N−acetylglucosamine−derived O−antigen building blocks biosynthesis
8.73 L−glutamate degradation VIII (to propanoate)
7.36 formaldehyde assimilation II (RuMP Cycle)
7.09 L−leucine degradation I
6.98 formaldehyde oxidation I
6.76 aromatic biogenic amine degradation
6.73 2−methylcitrate cycle I
6.44 mycolyl−arabinogalactan−peptidoglycan complex biosynthesis
6.39 UDP−2,3−diacetamido−2,3−dideoxy−α−D−mannuronate biosynthesis
6.38 chondroitin sulfate degradation I (bacterial)
6.38 dTDP−N−acetylviosamine biosynthesis
6.17 glycolysis V (Pyrococcus)
5.81 isoprene biosynthesis II (engineered)
5.8 protocatechuate degradation II (ortho−cleavage pathway)
5.63 adenosylcobalamin biosynthesis I (early cobalt insertion)
5.13 pyrimidine deoxyribonucleotides biosynthesis from CTP
4.67 pyrimidine deoxyribonucleotides de novo biosynthesis IV
3.14 methanogenesis from acetate
2.89 mycothiol biosynthesis
2.75 TCA cycle IV (2−oxoglutarate decarboxylase)
2.73 myo−inositol degradation I
2.7 mono−trans, poly−cis decaprenyl phosphate biosynthesis
2.66 pyrimidine deoxyribonucleotides de novo biosynthesis III
2.64 superpathway of glycolysis, pyruvate dehydrogenase, TCA, and glyoxylate bypass
2.31 myo−, chiro− and scillo−inositol degradation
2.18 L−arginine biosynthesis III (via N−acetyl−L−citrulline)
2.03 acetyl−CoA fermentation to butanoate II
log2FC
10
5
0
ARAS vs AR
8.71 L−leucine degradation I
8.15 2−methylcitrate cycle I
8.07 aromatic biogenic amine degradation
7.17 protocatechuate degradation II (ortho−cleavage pathway)
6.66 adenosylcobalamin biosynthesis I (early cobalt insertion)
5.5 pyrimidine deoxyribonucleotides biosynthesis from CTP
5.04 pyrimidine deoxyribonucleotides de novo biosynthesis IV
4 methanogenesis from acetate
log2FC
10
8
6
4
2
0
